# Supplementary material for: G9a and ZNF644 Physically Associate to Suppress Progenitor Gene Expression during Neurogenesis
Source: Stem Cell Reports. 2016 Aug 18;7(3):454–70. doi: 10.1016/j.stemcr.2016.06.012 (PMC5031922; doi:10.1016/j.stemcr.2016.06.012)
Supplement: Document S1. Supplemental Experimental Procedures and Figures S1–S6 [file mmc1.pdf]

Stem Cell Reports, Volume 7

Supplemental Information

G9a and ZNF644 Physically Associate to Suppress Progenitor Gene Expression during Neurogenesis

Jonathan B. Olsen, Loksum Wong, Steven Deimling, Amanda Miles, Hongbo Guo, Yue Li, Zhaolei Zhang, Jack F. Greenblatt, Andrew Emili, and Vincent Tropepe

SUPPLEMENTARY FIGURES AND TABLE LEGENDS

Figure S1. (A) Quantitative (q)PCR assays monitoring the relative expression level of ZNF644 mRNA from HEK293 cell lines stably expressing the indicated shRNA (n=3). Quantitation represented as mean ± SD. A non-silencing shRNA was used as a control. WISH assays monitoring the expression patterns of **(B)** *znf644a* and **(C)** *znf644b*. Both *znf644a* and *znf644b* are expressed maternally, and display widespread expression through the first 24hpf. At 48hpf expression is decreased, but still present in both anterior structures, and throughout the trunk. At each developmental stage no staining is apparent in sense strand control stained embryos. **(D)** RT-PCR assay monitoring the expression of *znf644a*, *znf644b* or β-actin cells from retinal extracts at the indicated time points.

Figure S2: (A) Lateral views of WT or *g9a* morphant embryos illustrating the varying degrees of developmental defects observed. **(B)** Frequency at which the normal/abnormal morphologies are observed in WT or the indicated g9a-MO injected embryos. The mismatch control MO (g9a-mMO) is indicated. **(C)** Lateral views of WT, *znf644a*, or *znf644b* morphant embryos at 48 hpf as well as rescue assays in which the respective cognate mRNA were co-injected. **(D)** The frequency at which the WT or abnormal phenotypes were observed in indicated embryos.

Figure S3. (A) WISH assays monitoring the expression of *vsx2* at 48 hpf or *otx2* at 72 hpf in lateral views or retinal cross-sections from WT, *znf644a* morphant or *znf644b* morphant embryos, as well as morphant embryos rescued by co-injection of the indicated mRNA. Red arrows indicate hindbrain neural cell populations that express *vsx2*. **(B)** Frequency at which the indicated embryos exhibited normal or mislocalized expression

of *vsx2* (top) or *otx2* (bottom). **(C)** WISH assays monitoring the expression of *lef1* at 48 hpf (dorsal views) in WT, *g9a* morphant, *znf644a* morphant, or *znf644b* morphant embryos. Red arrows point to *lef1*-positive midbrain regions, and red arrowheads point to *lef1*-expressing cells of the anterior hypothalamus. **(D)** ChIP-PCR assays monitoring the levels of H3K9me2 at the indicated positions near the TSS of *vsx2* or *ccnd1* genes (as in Figure 3C) at 48 hpf. An anti-H3K9me2 antibody and an isotype control (IgG) antibody were used for ChIP, followed by PCR amplification (35 cycles). Densitometry analysis revealed a ~50% reduction in the levels of H3K9me2 at the *ccnd1* promoter, and complete loss of H3K9me2 levels at the *vsx2* promoter in *g9a* morphant embryos. **(E)** Immunostaining with H3K9me2 antibody demonstrates a global reduction in nuclear expression in the *g9a* morphant retinas at 48 hpf compared to controls.

Figure S4. <u>Top</u>: **(A)** WISH assays monitoring the expression of *vsx2* or *ccnd1* at 48 hpf in WT or *g9a* morphant retinal cross-sections. **(B)** Immunostaining assays monitoring BrdU- or pH3-postive cells at 48 hpf in WT or *g9a* morphant retinal cross-sections. **(C)** Immunostaining assays monitoring the expression of the indicated neuronal markers in *g9a* morphant retinal cross-sections at the indicated time points. <u>Bottom</u>: Blastula cell transplantation experiments (n=3 separate experiments) of donor morphant cells to wildtype host cells. All transplanted embryos were screened at 24 hpf and only those embryos with GFP+ contribution to the retina were further analyzed. **(D-E)** Only 2 out of 30 positively screened transplanted embryos contained GFP+ znf644a-MO cells within the retina at 72 hpf. **(D)** One embryo demonstrated a relatively large population of GFP+ morphant cells near the retinal periphery, which is more permissive for proliferation. Many donor cells at the periphery were also pH3+, and donor morphant cells located at

the central retina were also proliferating ectopically (arrows). **(E)** The other embryo had very few cells in the center retina, but still showed evidence of ectopic proliferation (arrow). In both instances, host central retinal cells were not induced to proliferate. **(F)** Subphenotypic dose of GFP+ g9a-MO in donor cells does not lead to cell death after transplantation. **(G)** Subphenotypic dose of GFP+ znf644b-MO in donor cells does not lead to cell death after transplantation. **(H)** Combined subphenotypic doses of GFP+ g9a-MO and znf644a-MO results in donor cells undergoing cell death (caspase3+) that is confined to the donor cells. In each experiment, n>10 embryos were screened as GFP+ at 24 hpf, and n=3 were sectioned for analysis in each group at 48 hpf. Representative images of sections are shown. The WT *ccnd1* (48 hpf) image in Figure S4A are re-used from Figure 3D; the WT BrdU (48 hpf) in Figure S4B is re-used from Figure 4A; the WT GABA, PKC and Zpr1 (72 hpf) images in Figure S4C are re-used from Figure 4F; and the WT *vsx2* (48 hpf) images in Figure S4C are re-used from Figure 5A.

Figure S5. Top: CRISPR-Cas9 targeting of the *znf644b* gene show multiple mutated alleles in the F_1 generation that correlates with cellular defects in the retina. (**A**) PCR and restriction digest diagnostic on genomic DNA extracted from individual F_0 embryos injected with Cas9 mRNA and sgRNA targeting against exon 2 of *znf644b*. The PCR fragment contains two Bc1I digestion sites, one of which is located within the sgRNA-targeting site. Digestion of wild-type sequences results in three fragments, 449bp, 170bp and 144bp, whereas CRISPR-mediated insertion/deletion mutations result in loss of the digestion site in the target site, resulting in an additional 619bp fragment. (**B,** top) WISH assays monitoring the expression of ccnd1 in F_1 CRISPR embryos at 48hpf. One F1 embryo heterozygous for a frameshift mutation and was phenotypically normal for brain

and retinal growth as well as *ccnd1* expression at 48 hpf (embryo#1). A separate embryo with two mutant (frameshift) alleles showed significantly reduced brain and retinal growth and elevated expression of *ccnd1* (embryo#3), which was reminiscent of the *znf644b* morphant phenotype. (**C,** top) Immunostaining assay monitoring pH3-positive cells in retinal cross-sections from F_1 CRISPR embryos at 48hpf. One embryo had two *znf644b* mutant alleles, one of which was predicted to be a very large deletion likely resulting in a non-functional protein, while the other allele had a missense mutation leading to a predicted substitution of only two amino acids. The overall phenotype of this embryo was comparable to WT, suggesting that this embryo was functionally heterozygous (embryo#1). Other embryos genotyped as heteroallelic mutants displayed phenotypes that were similar to the morphants, such as a reduced retinal size and persistent pH3+ cells in the central retina at 48 hpf (embryo#2: 11.67 ± 4.16; embryo #3: 12.67 ± 2.08 compared to WT: 3.8 ± 0.5, n=9). (**B** & **C,** bottom) The corresponding sequencing data for both alleles of the embryos analyzed, with the predicted effect on the protein function. Black letters represent unchanged bases; Blue letters represent the PAM sequence; Red letters represent the target sequence; Pink letters represent the Bc1I digestion site; Dashes represent deletions; Green letters represent inserted bases. <u>Bottom</u>: Expression overlap of cell cycle regulator PCNA and the retinal differentiation marker Pax6 in the *znf644b* morphant retina. At 56 hpf, which coincides with the onset of widespread cell death of central retinal cells in *znf644b* morphants, PCNA can be found to be expressed in differentiated amacrine and ganglion cells (Pax6-postive) in the *znf644b* morphant retina, even though this population of neurons is reduced overall.

Figure S6. Interaction of *g9a* and *znf644* genes in regulating progenitor cell cycle. **(A)** WISH assays monitoring the expression of *ccnd1* in (top) midbrain or (bottom) retinal cross-sections at 48 hpf from WT embryos or embryos injected with the indicated combined subthreshold MO doses. **(B)** Immunostaining assays monitoring pH3 levels or BrdU incorporation at 48 hpf from WT or embryos injected with the indicated combined subthreshold MO doses. **(C)** Rescue experiments in which mRNA encoding WT human (ZNF644) or mutant version lacking G9a/GLP binding capability (C1263A) is co-injected with znf644a-MO (left) or znf644b-MO (right). *Vsx2* expression was assessed by WISH at 48 hpf, and PCNA, pH3, and Zpr1 levels (n=3 in each group) were assessed by immunostaining and confocal microscopy at the indicated time points.

Table S1. LC-MS/MS dataset for 33 replicate AP-MS experiments for histone methylation- and transcription-related proteins. Bait-prey interactions are indicated, as are the interaction scores based on a SAINT cut-off of ≥ 0.83.

Table S2. Dataset for replicate AP-MS assays using GFP-tagged ZNF644. Bait proteins and replicate number are indicated in column headers, with identified prey proteins as row headers. Numerical values represent spectral counts based on LC-MS/MS data.

Tables S3-S4. WT vs znf644a-MO (S3) and WT vs znf644b-MO. Positioning of H3K9me2 peaks from ChIP-seq in znf644a-MO or znf644b-MO relative WT embryos at 48 hpf.

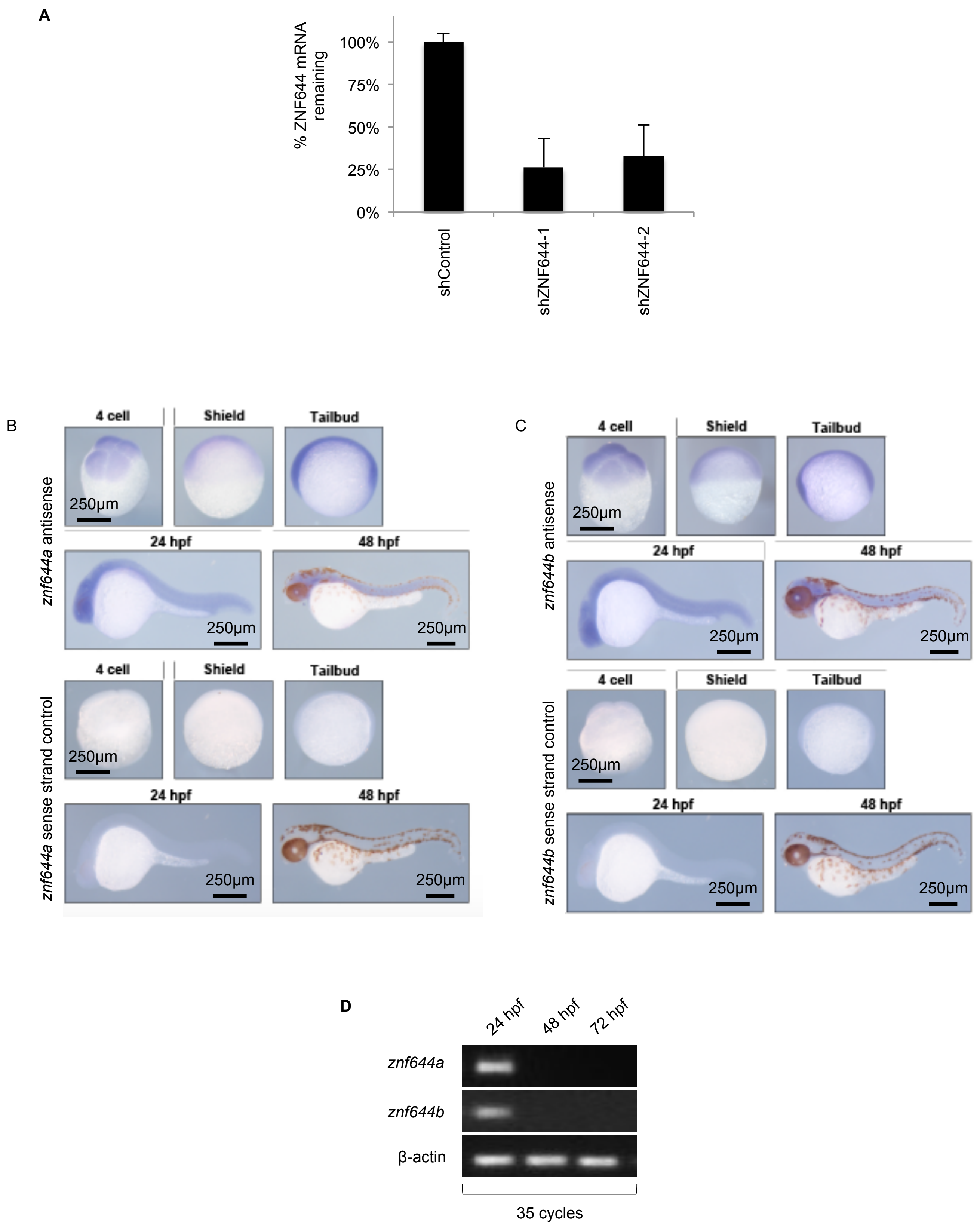

A
% ZNF644 mRNA remaining
100%
75%
50%
25%
0%
shControl
shZNF644-1
shZNF644-2
B
znf644a antisense
4 cell
Shield
Tailbud
24 hpf
48 hpf
250μm
znf644a sense strand control
C
znf644b antisense
znf644b sense strand control
D
24 hpf
48 hpf
72 hpf
znf644a
znf644b
β-actin
35 cycles

Figure S1

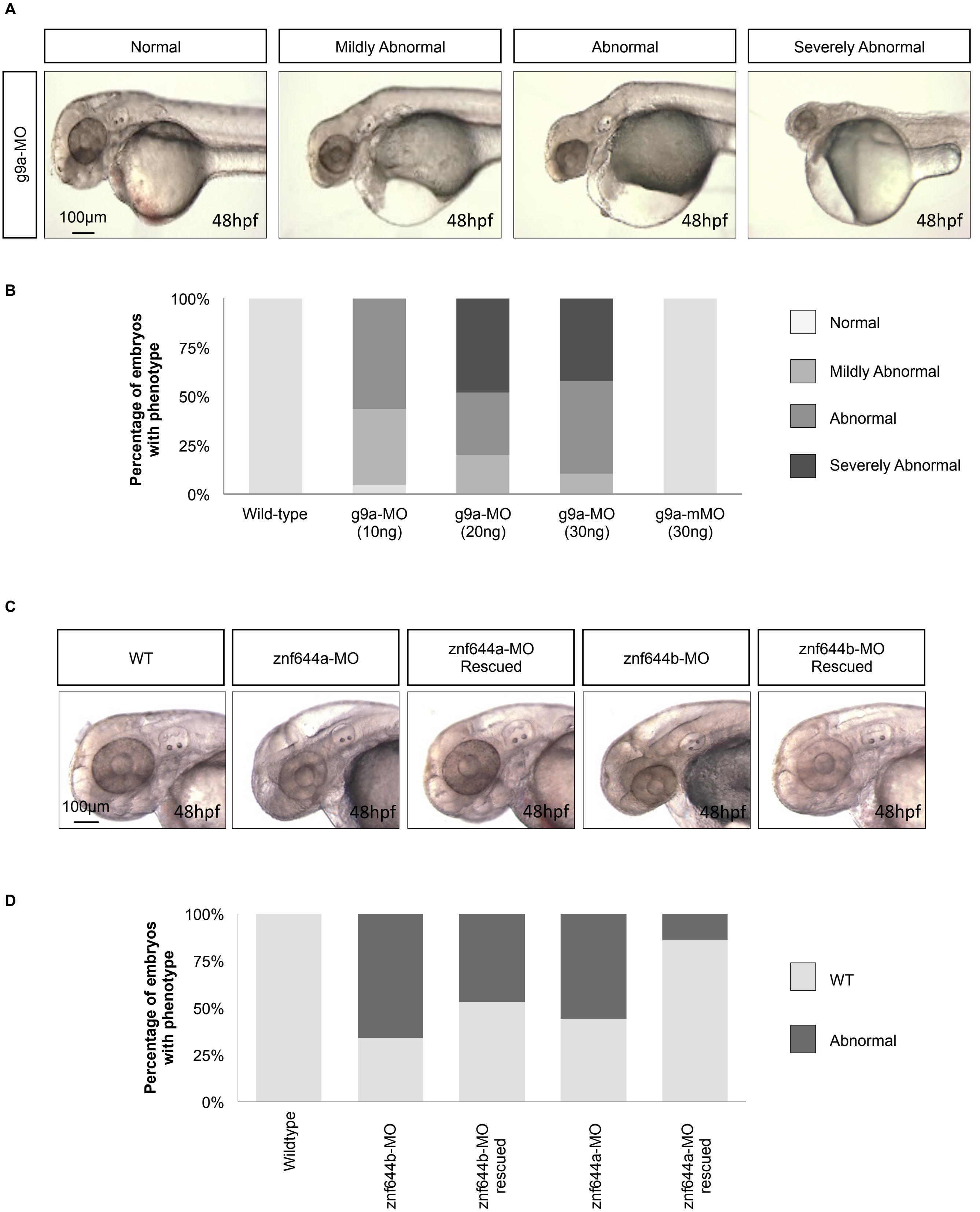
A
Normal
Mildly Abnormal
Abnormal
Severely Abnormal
g9a-MO
100µm
48hpf
48hpf
48hpf
48hpf
B
Percentage of embryos with phenotype
100%
75%
50%
25%
0%
Wild-type
g9a-MO (10ng)
g9a-MO (20ng)
g9a-MO (30ng)
g9a-mMO (30ng)
Normal
Mildly Abnormal
Abnormal
Severely Abnormal
C
WT
znf644a-MO
znf644a-MO Rescued
znf644b-MO
znf644b-MO Rescued
100µm
48hpf
48hpf
48hpf
48hpf
48hpf
D
Percentage of embryos with phenotype
100%
75%
50%
25%
0%
Wildtype
znf644b-MO
znf644b-MO rescued
znf644a-MO
znf644a-MO rescued
WT
Abnormal

Figure S2

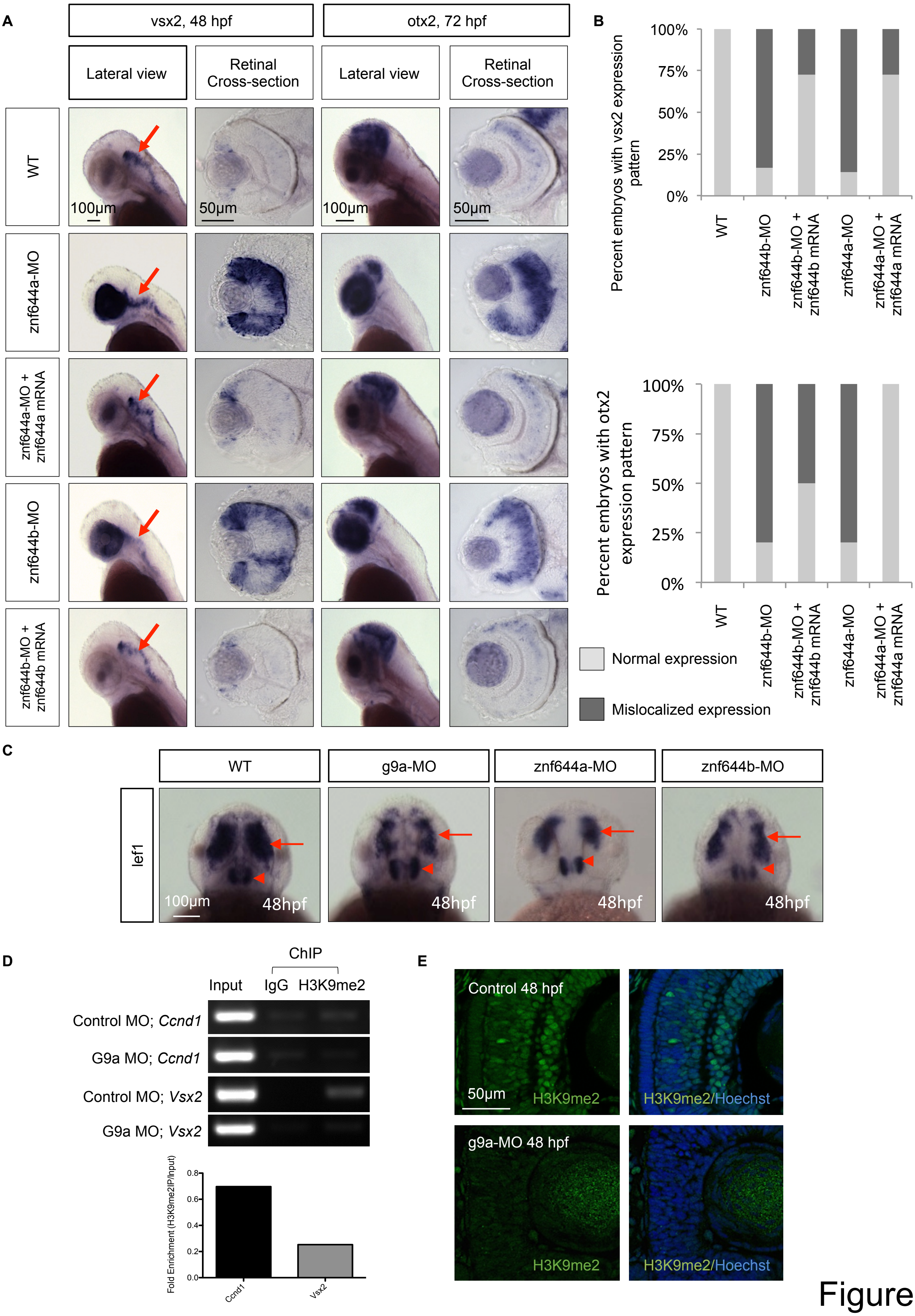
A
vsx2, 48 hpf
otx2, 72 hpf
Lateral view
Retinal Cross-section
Lateral view
Retinal Cross-section
WT
znf644a-MO
znf644a-MO + znf644a mRNA
znf644b-MO
znf644b-MO + znf644b mRNA
100µm
50µm
100µm
50µm
B
Percent embryos with vsx2 expression pattern
100%
75%
50%
25%
0%
WT
znf644b-MO
znf644b-MO + znf644b mRNA
znf644a-MO
znf644a-MO + znf644a mRNA
Percent embryos with otx2 expression pattern
Normal expression
Mislocalized expression
C
WT
g9a-MO
znf644a-MO
znf644b-MO
lef1
100µm
48hpf
D
ChIP
Input
IgG
H3K9me2
Control MO; Ccnd1
G9a MO; Ccnd1
Control MO; Vsx2
G9a MO; Vsx2
Fold Enrichment (H3K9me2IP/Input)
0.8
0.6
0.4
0.2
0.0
Ccnd1
Vsx2
E
Control 48 hpf
g9a-MO 48 hpf
50µm
H3K9me2
H3K9me2/Hoechst

Figure S3

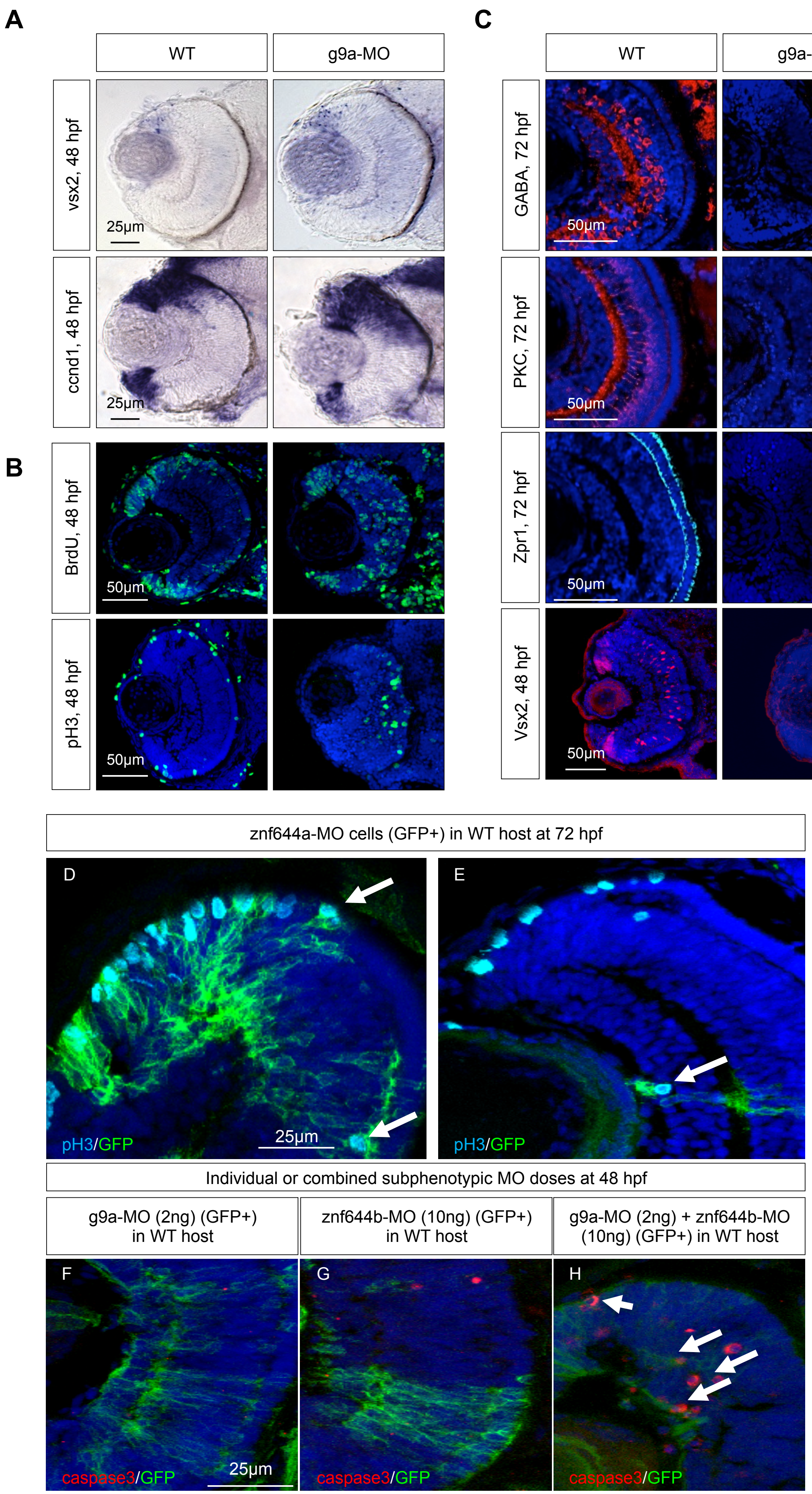
A
WT
g9a-MO
vsx2, 48 hpf
25µm
ccnd1, 48 hpf
25µm
B
BrdU, 48 hpf
50µm
pH3, 48 hpf
50µm
C
WT
g9a-MO
GABA, 72 hpf
50µm
PKC, 72 hpf
50µm
Zpr1, 72 hpf
50µm
Vsx2, 48 hpf
50µm
znf644a-MO cells (GFP+) in WT host at 72 hpf
D
pH3/GFP
25µm
E
pH3/GFP
Individual or combined subphenotypic MO doses at 48 hpf
g9a-MO (2ng) (GFP+) in WT host
znf644b-MO (10ng) (GFP+) in WT host
g9a-MO (2ng) + znf644b-MO (10ng) (GFP+) in WT host
F
caspase3/GFP
25µm
G
caspase3/GFP
H
caspase3/GFP

Figure S4

A

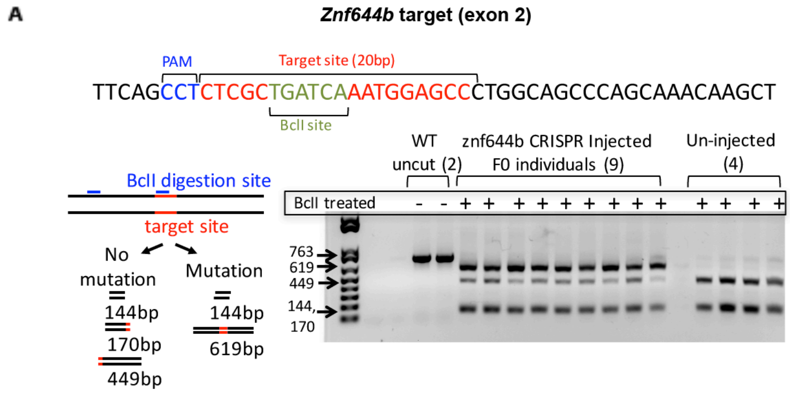

B

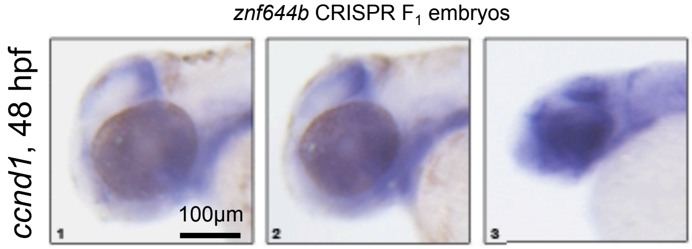

	INDEL	EFFECT ON PROTEIN
WT Allele: TTCAGCCTCTCGCTGATCAAATGGAGCCCTGGCAGCCCAGCAAACAAGCT		
1-Allele 1: TTCAGCCTCTCGCTGATCAAATGGAGCCCTGGCAGCCCAGCAAACAAGCT	0	NO EFFECT
1-Allele 2: TTCAGC - - - - - - - TGATCAAATGGAGCCCTGGCAGCCCAGCAAACAAGCT	-7	FRAMESHIFT
2-Allele 1: TTCAGCCTCTC - - - - - - - AATGGAGCCCTGGCAGCCCAGCAAACAAGCT	-7	FRAMESHIFT
2-Allele 2: TTCAGCC - - - - - GT - - - - - - AAATGGAGCCCTGGCAGCCCAGCAAACAAGCT	-11, +2	MISSENSE MUTATION
3-Allele 1: TTCAGCCTCTC - - - - - TCAAATGGAGCCCTGGCAGCCCAGCAAACAAGCT	-5	FRAMESHIFT
3-Allele 2: TTCAGCCTCT - - - - GATCAAATGGAGCCCTGGCAGCCCAGCAAACAAGCT	-4	FRAMESHIFT

C

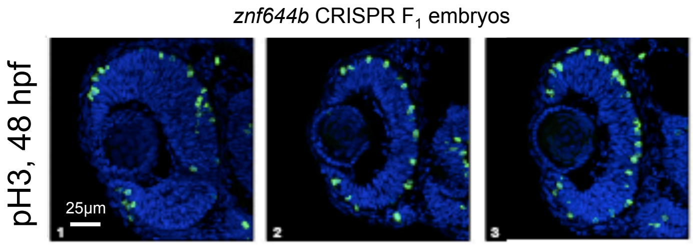

	INDEL	EFFECT ON PROTEIN
WT Allele: TTCAGCCTCTCGCTGATCAAATGGAGCCCTGGCAGCCCAGCAAACAAGCT		
1-Allele 1: TTCAGCCTGAAATGGATCAAATGGAGCCCTGGCAGCCCAGCAAACAAGCT	-6, +6	MISSENSE
1-Allele 2: UNKNOWN LARGE DELETION	>310	LARGE DELETION
2-Allele 1: TTCAGCCTCTCCAAAGCTGATCAAATGGAGCCCTGGCAGCCCAGCAAACAAGCT	+4	FRAMESHIFT
2-Allele 2: TTCAGCCTCTC - - - - - - - TCGGATGC - - - - - - AAATGGAGCCCTGGCAGCCCAGCAA	-13, +8	FRAMESHIFT
3-Allele 1: TTCAGCCTCT - - - - - ATCAAATGGAGCCCTGGCAGCCCAGCAAACAAGCT	-5	FRAMESHIFT
3-Allele 2: TTCAGCCT - - - - GCCGGAGCC - - - - TCAAATGGAGCCCTGGCAGCCCAGCAAACAA	-8, +9	FRAMESHIFT

E

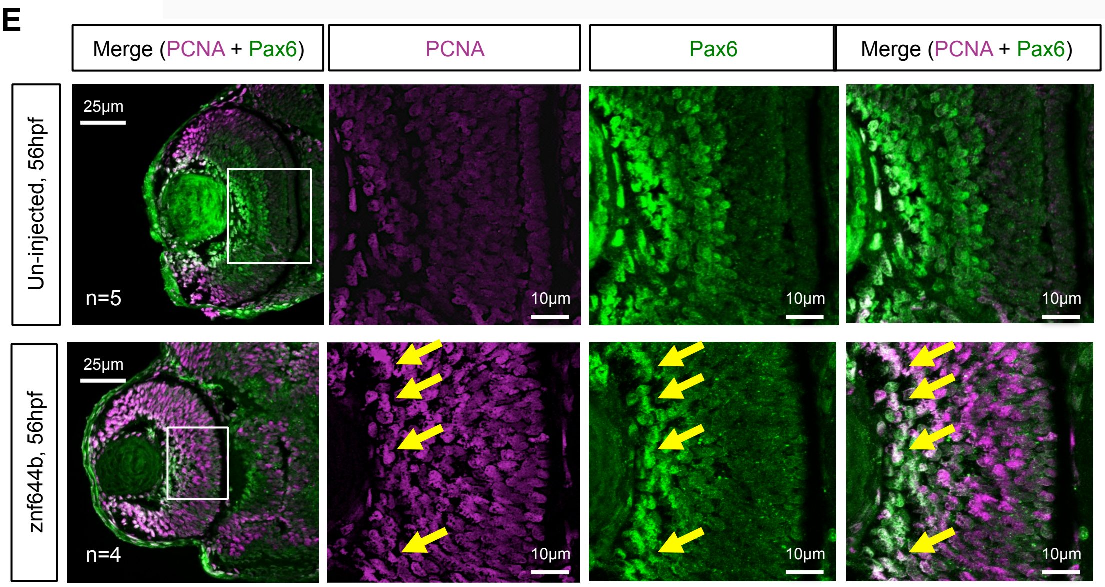

Figure S5

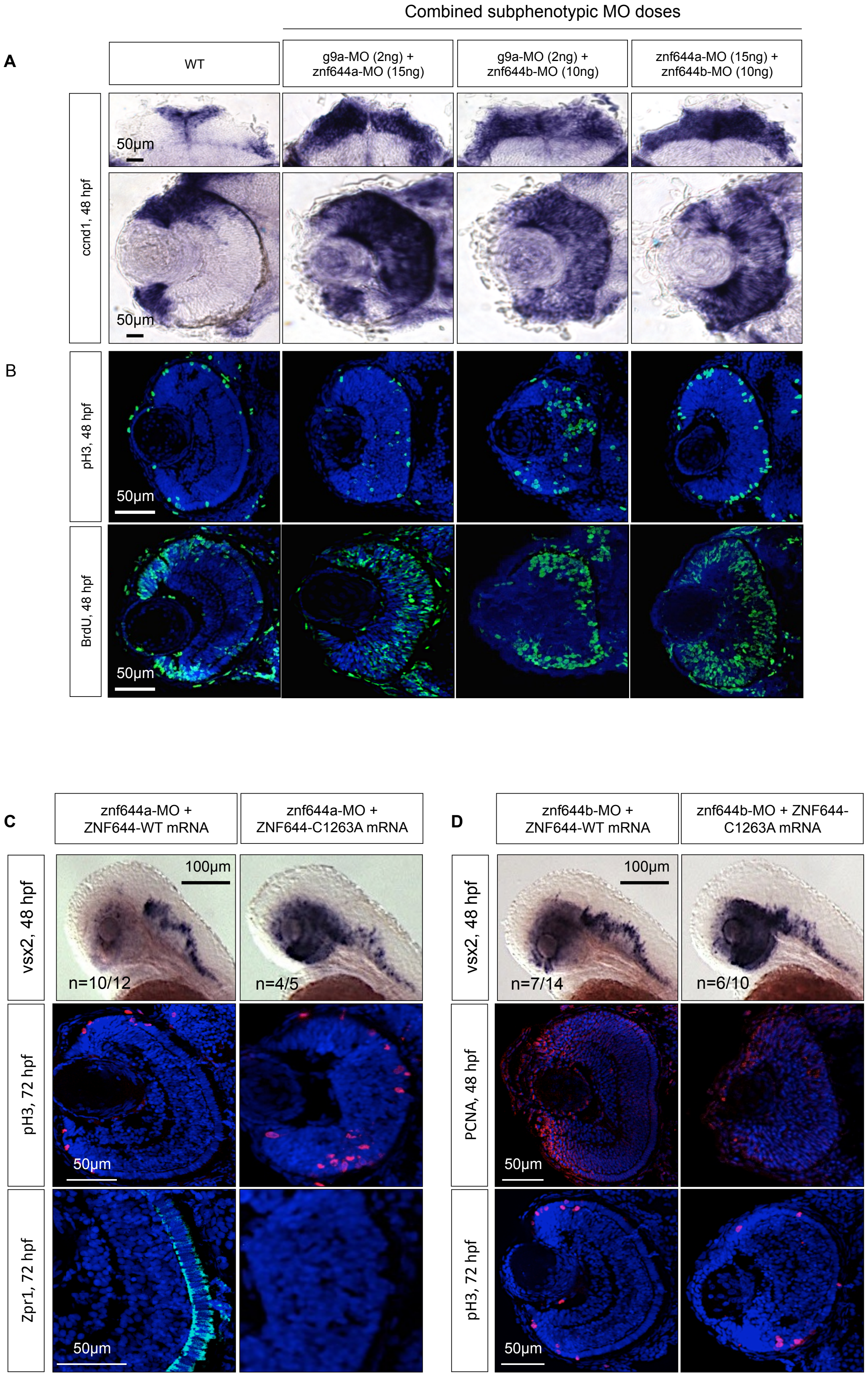
Combined subphenotypic MO doses
A
WT
g9a-MO (2ng) + znf644a-MO (15ng)
g9a-MO (2ng) + znf644b-MO (10ng)
znf644a-MO (15ng) + znf644b-MO (10ng)
ccnd1, 48 hpf
50µm
50µm
B
pH3, 48 hpf
50µm
BrdU, 48 hpf
50µm
C
znf644a-MO + ZNF644-WT mRNA
znf644a-MO + ZNF644-C1263A mRNA
vsx2, 48 hpf
100µm
n=10/12
n=4/5
pH3, 72 hpf
50µm
Zpr1, 72 hpf
50µm
D
znf644b-MO + ZNF644-WT mRNA
znf644b-MO + ZNF644-C1263A mRNA
vsx2, 48 hpf
100µm
n=7/14
n=6/10
PCNA, 48 hpf
50µm
pH3, 72 hpf
50µm

Figure S6

SUPPLEMENTAL METHODS

RNA interference and western blotting

HEK293 cells stably-expressing shRNA targeting Luciferase (non-silencing control), G9a or ZNF644 were generated as previously described (Mak et al., 2010). shRNA clones (kind gifts from Jason Moffat) are as follows: shControl (TRCN0000072256), shG9a-1 (TRCN0000115667), shG9a-2 (TRCN0000115668), shZNF644-1 (TRCN0000140402), and shZNF644-2 (TRCN0000145205). The following antibodies were used for immunodetection: rabbit anti-G9a (C6H3, Cell Signalling, #3306), rabbit anti-H3K9me2 (D85B4, Cell Signalling, #4658), rabbit anti-H3K9me3 (AbCam, ab8898), mouse anti-GAPDH (6C5, AbCam, ab8245), and rabbit anti-H3 antibody (AbCam, ab1791). Validation of shRNA-mediated knockdown of ZNF644 was determined by qPCR and quantified by the ΔΔCt method.

Morpholino injections

Morpholinos doses used in this study are as follows: g9a-MO and control MO (Rai et al., 2010), 4ng; znf644a-MO (ATTAAAATTGTCACCTGTTTTGACT) and control MO (ATTTAATTTGTCAGCTGTATTCACT), 20ng; and znf644b-MO (GTGAGCAATAATCACCTTTTCTGAT) and control MO (GTGTGCATAATCTCCTTATCAGAT), 30ng. p53-MO was used as previously described (Robu et al., 2007).

Whole-mount in situ hybridization (WISH)

WISH was carried out as previously described (Deimling et al., 2015) with the following modifications: Embryos were digested in 5µg/ml Proteinase K and re-fixed for 20 min in 4% paraformaldehyde; TTw with 2mg/ml BSA was used in place of MAB on days 2 and 3, and

embryos were incubated in TTw-BSA overnight on day 3 before staining on day 4. Samples were washed out of glycerol and into sucrose gradients for cryosectioning as described (Wong et al., 2010).

ChIP-PCR and ChIP-seq analysis

ChIP-PCR primers were as follows (5'→3'): vsx2-F, AGACATTTTCCAGCGCACTT; vsx2-R, GCAATACGCATGATCCCTCT; ccnd1-F, TTCACCCCAGTCTTTTCCAC; and ccnd1-R, AAAGTCTCGCTGCAGCTCTC-3'. Alignments were performed by Bowtie2 (Langmead and Salzberg, 2012) and peak calling by MACS2 (Feng et al., 2012). Promoter regions of *vsx2* and *ccnd1* were amplified by PCR (35 cycles).

Immunostaining and BrdU incorporation assays

The following antibodies were used at the indicated dilutions: Caspase3 (cleaved, Asp175; Cell Signaling Technology, #9961), H3K9me2 (AbCam, ab1220) Pax6a (Covance, PRB-278P), PCNA (ZYMED Laboratories, 13-3900), pH3 (D2V8, Cell Signaling Technology, #3377), PKC (Santa Cruz, sc-209), Zn5 (ZIRC), Zpr1 (ZIRC), GABA (Sigma-Aldrich), anti-rabbit Cy3 (Jackson ImmunoResearch Laboratories, #111-165-003), anti-mouse Cy3 (Jackson ImmunoResearch Laboratories, #115-165-146), anti-mouse Cy5 (Jackson ImmunoResearch Laboratories, #115-165-146), and Vsx2 (a kind gift from Shin-ichi Higashijima).

Blastula transplantations

Donor cells from *Tg(β-actin:mGFP)* embryos injected with g9a-MO, znf644a-MO or znf644b-MO were transplanted into AB strain host embryos between 3-4 hpf. Only hosts with GFP+ retinal cells were cryosectioned for analysis using immunostaining.

CRISPR-Cas9 Mutagenesis

Targeting sites for CRISPR-Cas9 mediated mutagenesis were designed against znf644b using CHOPCHOP (https://chopchop.rc.fas.harvard.edu/index.php). The target site used for znf644b was GGCTCCATTTGATCAGCGAG. The single guide RNA (sgRNA) was transcribed directly from long oligonucleotides containing a T7 promoter, targeting sequence, and guide RNA sequence using MEGAshortscript (Ambion AM1354). The sgRNA was purified using ethanol/ammonium acetate precipitation. Cas9 mRNA was transcribed from XbaI-linearized pT3TS-nCas9n plasmid using T3 MEGAscript kit (Ambion AM1338) and purified using lithium chloride precipitation. sgRNA (800pg) and Cas9 (600pg) mRNA were co-injected into the cell of 1-cell staged zebrafish embryos. Genomic DNA from a portion of injected embryos was individually extracted and a region around the target site was PCR amplified (F primer TGTGCCAAGACTGAAGACTGTT; R primer AGTCATCTCCCTCTTGTGGTGT), purified and digested with the restriction enzyme Bc1I (NEB) to assess for mutations. The remaining embryos were raised to adulthood. Once adulthood was reached, the fish were in-crossed and similarly assessed for germ-line transmitted mutations in the F_1 progeny via the restriction digest diagnostic. The DNA extracted from individual F_1 embryos was also sequenced to confirm the extent and type of mutation. To correlate genotype to phenotype the gDNA was extracted from the tail of the embryos, PCR and sequenced, while the head was used for in situ hybridization and immunohistochemistry.

MASS SPECTROMETRY AND DATA ANALYSIS

After identification of interacting proteins using a precision hybrid Orbitrap-Velos mass spectrometer (ThermoFisher Scientific)(Walkey et al., 2012), confidence values were assigned to

protein-protein interactions using a strict Statistical Analysis of Interactome (Choi et al., 2011) algorithm cut-off score of ≥0.83.

REFERENCES

Choi, H., Larsen, B., Lin, Z.Y., Breitkreutz, A., Mellacheruvu, D., Fermin, D., Qin, Z.S., Tyers, M., Gingras, A.C., and Nesvizhskii, A.I. (2011). SAINT: probabilistic scoring of affinity purification-mass spectrometry data. Nature methods *8*, 70-73.

Deimling, S.J., Halabi, R.R., Grover, S.A., Wang, J.H., and Drysdale, T.A. (2015). Understanding early organogenesis using a simplified in situ hybridization protocol in Xenopus. Journal of visualized experiments : JoVE, e51526.

Feng, J., Liu, T., Qin, B., Zhang, Y., and Liu, X.S. (2012). Identifying ChIP-seq enrichment using MACS. Nature protocols *7*, 1728-1740.

Langmead, B., and Salzberg, S.L. (2012). Fast gapped-read alignment with Bowtie 2. Nature methods *9*, 357-359.

Mak, A.B., Ni, Z., Hewel, J.A., Chen, G.I., Zhong, G., Karamboulas, K., Blakely, K., Smiley, S., Marcon, E., Roudeva, D.*, et al.* (2010). A lentiviral functional proteomics approach identifies chromatin remodeling complexes important for the induction of pluripotency. Molecular & cellular proteomics : MCP *9*, 811-823.

Rai, K., Jafri, I.F., Chidester, S., James, S.R., Karpf, A.R., Cairns, B.R., and Jones, D.A. (2010). Dnmt3 and G9a cooperate for tissue-specific development in zebrafish. The Journal of biological chemistry *285*, 4110-4121.

Robu, M.E., Larson, J.D., Nasevicius, A., Beiraghi, S., Brenner, C., Farber, S.A., and Ekker, S.C. (2007). p53 activation by knockdown technologies. PLoS genetics *3*, e78.

Walkey, C.D., Olsen, J.B., Guo, H., Emili, A., and Chan, W.C. (2012). Nanoparticle size and surface chemistry determine serum protein adsorption and macrophage uptake. Journal of the American Chemical Society *134*, 2139-2147.

Wong, L., Weadick, C.J., Kuo, C., Chang, B.S., and Tropepe, V. (2010). Duplicate dmbx1 genes regulate progenitor cell cycle and differentiation during zebrafish midbrain and retinal development. BMC developmental biology *10*, 100.
