# Supplementary material for: G9a and ZNF644 Physically Associate to Suppress Progenitor Gene Expression during Neurogenesis
Source: Stem Cell Reports. 2016 Aug 18;7(3):454–70. doi: 10.1016/j.stemcr.2016.06.012 (PMC5031922; doi:10.1016/j.stemcr.2016.06.012)
Supplement: Document S2. Article plus Supplemental Information [file mmc6.pdf]

Stem Cell Reports
Article
OPEN ACCESS

G9a and ZNF644 Physically Associate to Suppress Progenitor Gene Expression during Neurogenesis

Jonathan B. Olsen,[1,2,7] Loksum Wong,[3,7] Steven Deimling,[3] Amanda Miles,[3] Hongbo Guo,[1] Yue Li,[1,2,6] Zhaolei Zhang,[1,2,6] Jack F. Greenblatt,[1,2] Andrew Emili,[1,2,*] and Vincent Tropepe[3,4,5,*]
[1]Donnelly Centre for Cellular and Biomolecular Research, University of Toronto, Toronto, ON M5S 3E1, Canada
[2]Department of Molecular Genetics, University of Toronto, Medical Science Building, Toronto, ON M5S 3E1, Canada
[3]Department of Cell and Systems Biology, University of Toronto, Toronto, ON, M5S 3G5, Canada
[4]Department of Ophthalmology and Vision Sciences, University of Toronto, Toronto, ON M5T 3A9, Canada
[5]Centre for the Analysis of Genome Evolution and Function, University of Toronto, Toronto, ON M5S 3B2, Canada
[6]Department of Computer Science, University of Toronto, Toronto, ON M5S 3G4, Canada
[7]Co-first author
*Correspondence: v.tropepe@utoronto.ca (V.T.), andrew.emili@utoronto.ca (A.E.)
http://dx.doi.org/10.1016/j.stemcr.2016.06.012

SUMMARY

Proliferating progenitor cells undergo changes in competence to give rise to post-mitotic progeny of specialized function. These cell-fate transitions typically involve dynamic regulation of gene expression by histone methyltransferase (HMT) complexes. However, the composition, roles, and regulation of these assemblies in regulating cell-fate decisions in vivo are poorly understood. Using unbiased affinity purification and mass spectrometry, we identified the uncharacterized C2H2-like zinc finger protein ZNF644 as a G9a/GLP-interacting protein and co-regulator of histone methylation. In zebrafish, functional characterization of ZNF644 orthologs, *znf644a* and *znf644b*, revealed complementary roles in regulating G9a/H3K9me2-mediated gene silencing during neurogenesis. The non-overlapping requirements for *znf644a* and *znf644b* during retinal differentiation demarcate critical aspects of retinal differentiation programs regulated by differential G9a-ZNF644 associations, such as transitioning proliferating progenitor cells toward differentiation. Collectively, our data point to ZNF644 as a critical co-regulator of G9a/H3K9me2-mediated gene silencing during neuronal differentiation.

INTRODUCTION

Alterations in histone methylation instruct developmental gene-expression programs that enable proliferating progenitor cells to exit the cell cycle and differentiate. These changes are mediated by conserved, multiprotein macromolecules that "write" and "read" histone methylation marks, such as Set1/COMPASS-like complexes (Shilatifard, 2012), Polycomb repressor complexes (Margueron and Reinberg, 2011), and assemblies containing the histone methyltransferases (HMTs) G9a and GLP (Shinkai and Tachibana, 2011). Developmental regulation of specific genomic loci involves complex physical interactions involving tissue-specific transcription factors (TFs), non-coding RNA, and other co-factors (Guttman et al., 2011). Histone methylation in pluripotency-related gene regulation has been characterized extensively (Watanabe et al., 2013), yet the composition of relevant HMT complexes and, specifically, the identity of physically associated co-regulators that modulate activity during cellular differentiation are incompletely defined.

G9a/EHMT2 and GLP/EHMT1 are responsible for dimethylated H3K9 (H3K9me2) in transcriptionally repressed euchromatin (Tachibana et al., 2005) and are essential for cell differentiation during embryogenesis (Shinkai and Tachibana, 2011). In embryonic stem cells (ESCs), G9a/GLP facilitate the long-term silencing of pluripotency-associated genes (Tachibana et al., 2008). In hematopoietic stem and progenitor cells (HSPCs), inhibition of G9a/GLP delays lineage commitment and prevents the formation of large H3K9me2 chromatin territories (Chen et al., 2012). In neural contexts, loss of G9a or GLP in the mouse forebrain reactivates neural progenitor gene expression, leading to cognitive and adaptive behavioral defects (Schaefer et al., 2009). Disruption of the GLP/*EHMT1* gene in humans is associated with congenital intellectual disability (Kleefstra et al., 2005), and heterozygous GLP/*EHMT1* knockout mice exhibit behavioral and neurodevelopmental abnormalities (Balemans et al., 2010, 2014). Although G9a/GLP-associated proteins have been reported (Bian et al., 2015; Maier et al., 2015; Ueda et al., 2006), their precise contributions to G9a/GLP-mediated neural differentiation are largely unknown.

Retinal progenitor cells (RPCs) are proliferative multipotent cells that produce differentiated cells in an evolutionary conserved birth order (Bassett and Wallace, 2012; Cepko et al., 1996). RPC proliferation and differentiation must be closely integrated and coordinated with eye growth for proper morphology and structure (Green et al., 2003; Wong et al., 2015), with impaired proliferative capacity resulting in microphthalmia, degeneration, and visual impairment (Levine and Green, 2004). RPC differentiation requires G9a/H3K9me2-mediated suppression of genes that sustain a proliferative multipotent state (Katoh

This is an open access article under the CC BY license (http://creativecommons.org/licenses/by/4.0/).

et al., 2012), including Vsx2/Chx10, a TF uniformly expressed in fully multipotent RPCs (Burmeister et al., 1996; Duffy et al., 2005; Vitorino et al., 2009), and Ccnd1/Cyclin D1, the predominant D-cyclin in the developing retina (Barton and Levine, 2008). Co-regulators that facilitate G9a/H3K9me2-mediated silencing during neurogenesis are unknown.

High-grade myopia involves progressive axial elongation of the eye that predisposes to degeneration and blindness. Genetic factors linked to high-grade myopia (Hawthorne and Young, 2013) include mutations in the C2H2-like zinc finger (ZF) protein ZNF644, which segregates with autosomal dominant inheritance (Shi et al., 2011). These findings suggest a role for ZNF644 in maintaining proper eye morphology and/or growth, yet its function in neural contexts is currently uncharacterized.

To better understand the molecular basis of histone methylation, we applied a lentiviral-based affinity purification and mass spectrometry (AP-MS) approach to isolate protein complexes that "write" and/or "read" histone methylation. We found that ZNF644 physically interacts with G9a and GLP and serves as a co-regulator of H3K9me2. By characterizing zebrafish ZNF644 orthologs *znf644a* and *znf644b*, we pinpointed roles for these genes in regulating G9a/H3K9me2-mediated silencing in neural progenitor cells. Loss-of-function experiments demonstrated roles for *znf644a* and *znf644b* in maintaining cell viability and ensuring the proper differentiation of retinal neurons, respectively, both of which were dependent on functional cooperativity and physical binding to G9a. Additional evidence suggested that the functions of *g9a* and *znf644a/b* are recapitulated in midbrain progenitor cells, suggesting a common gene-silencing complex mediating differentiation in distinct neuronal progenitor populations. Collectively, our findings highlight G9a-ZNF644 as a critical regulator of gene silencing and cell-fate transitions during neural differentiation.

RESULTS

ZNF644 Is a Co-regulator of Histone Methylation

We previously described a systematic AP-MS approach, based on stable lentivirus-based expression of epitope-tagged proteins, to characterize stable protein complexes involved in chromatin and transcriptional regulation (Mak et al., 2010; Ni et al., 2011). As part of a larger study (Marcon et al., 2014), we targeted human HMT protein complexes linked to histone methylation during development. These included confirmed and putative homologs of Polycomb, Set1/COMPASS, and G9a/GLP-containing protein complexes (Table S1). We created a scored physical interaction map encompassing components of histone methylation-related protein complexes with emphasis on regulators of developmental gene expression. Our dataset comprised 33 replicate AP-MS analyses of 25 histone methylation-related and eight transcription-related proteins.

In addition to accurately recapitulating established transcriptional assemblies, such as Mediator (Conaway et al., 2005), RNA polymerase II (RNAPII) (Ni et al., 2011), and cleavage and polyadenylation-specific termination factor (Kaufmann et al., 2004) (Figure 1 and Table S1), we identified dozens of previously unknown associations among a filtered network of 573 interactions. Pairwise hierarchical clustering of the highly correlated interaction profiles of the different bait proteins revealed components residing within the same complex(es) (Figure 1B). Most (312 of 428, 72.9%) of the annotated interactions detected were corroborated in public protein-protein interaction repositories such as BioGRID (Chatr-Aryamontri et al., 2015) and iRefWeb (Turner et al., 2010), suggesting a high-confidence interaction network.

In addition to its known dimerization partner GLP, affinity-tagged G9a reproducibly co-purified the C2H2-like ZF proteins, WIZ and ZNF644 (Figure 1C). WIZ was previously reported to interact with G9a/GLP in ESCs via association of the SET domains of G9a/GLP with an atypical C2H2-like ZF motif in WIZ (Ueda et al., 2006). ZNF644 contains a highly similar atypical C2H2-like ZF motif, and GFP-tagged full-length ZNF644 as well as an isoform comprising the atypical ZF region (ZF8) was sufficient to co-purify G9a/GLP and WIZ (Figure 1D). In addition, mutations in the putative zinc ion-chelating residues of the atypical ZF motif (C1263A or H1283A) abolished complex formation (Figure 1D). The shortened ZF8 region of ZNF644, expressed as a recombinant glutathione S-transferase (GST)-fusion protein, efficiently pulled down recombinant His-tagged G9a SET domain homodimer, but not the C1263A or H1283A mutants (Figure 1E). Small hairpin RNA (shRNA)-mediated knockdown of either G9a or ZNF644 significantly and specifically reduced H3K9me2 (Figures 1F and S1A). Collectively, these data demonstrate that ZNF644 binds to G9a/GLP and co-regulates H3K9me2.

znf644a and *znf644b* Are Expressed in Zebrafish Retina and Midbrain Progenitors

HMT-related proteins are widely conserved (Pu et al., 2010), allowing functional evaluation in model organisms. In zebrafish, *g9a* is broadly expressed during embryogenesis, and antisense splice-block morpholinos (MOs; g9a-MO) disrupting its function cause severe morphological defects, including reductions in eye and brain size (Rai et al., 2010). We identified two zebrafish ZNF644 orthologs with 19.6% and 27.1% amino acid identity, *znf644a* and *znf644b*, respectively (Figure 2A), that contain atypical C2H2-like ZF motifs, suggesting conserved physical association with

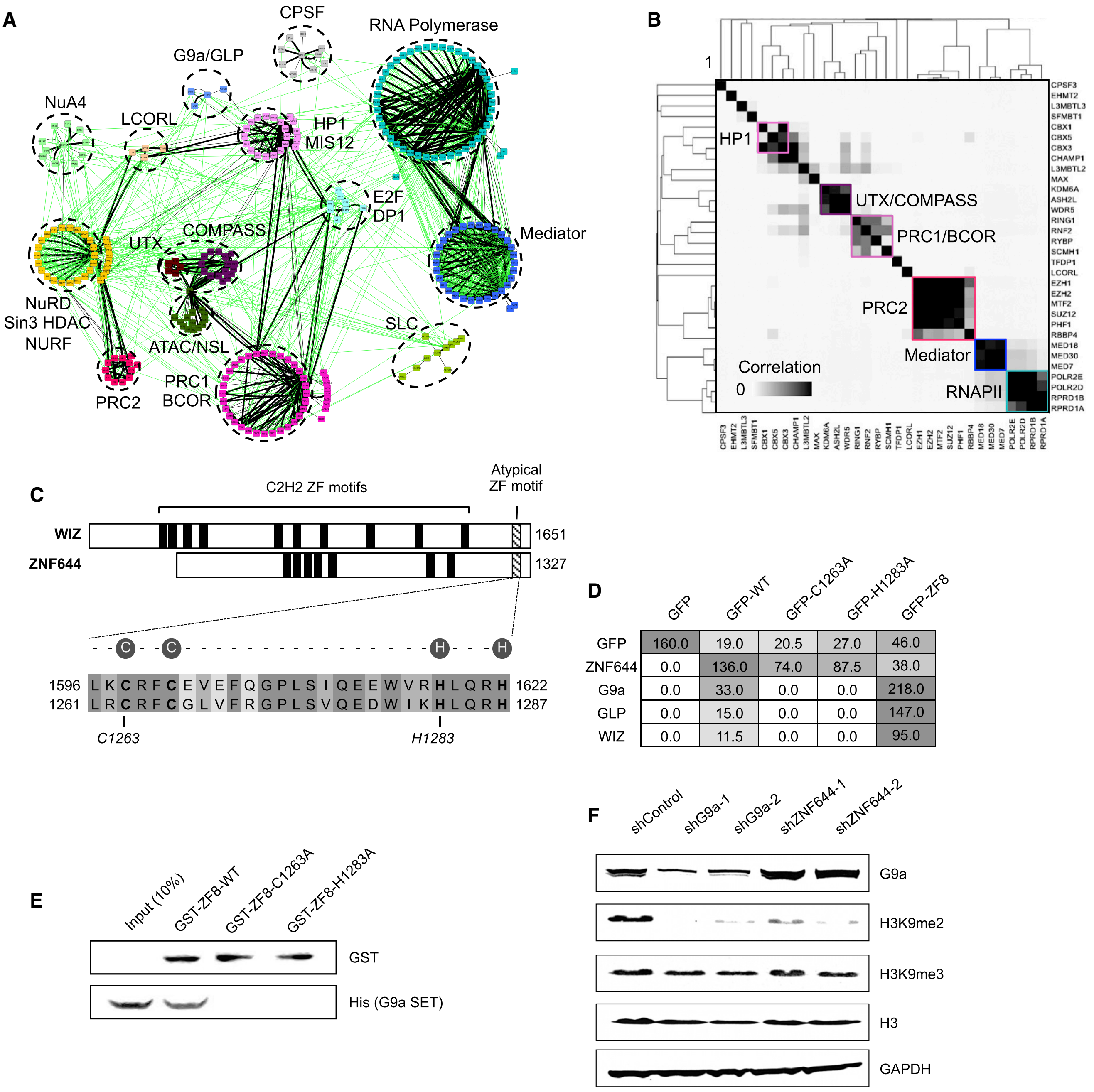

Figure 1. ZNF644 Is a G9a/GLP-Interacting Protein and Co-regulator of H3K9me2

(A) Physical interaction map of histone methylation-related complexes. Nodes represent proteins identified by AP-MS, black edges represent high-confidence interactions, and known interactions are in green.

(B) Hierarchical clustering of bait interaction profile similarity. Colored boxes indicate proteins known to reside in the same complex.

(C) (Top) Protein domain architecture of WIZ and ZNF644. (Bottom) Sequence alignment of atypical C2H2-like ZF motifs. Putative zinc ion-chelating residues are circled.

(D) Summary of AP-MS analyses showing average peptide spectral counts of GFP-tagged forms of full-length WT ZNF644, the C1263A or H1283A point mutants, or the ZF8-containing C-terminal region only.

(E) Western blot analysis of pulldown assays using GST-tagged WT, C1263A, or H1283A versions of the ZF8 domain of ZNF644 with the His-tagged G9a SET domain.

(F) Western blot assays monitoring G9a, H3K9me2, H3K9me3, histone H3, and GAPDH levels in HEK293 cell lines expressing shRNAs targeting a non-silencing control, G9a, or ZNF644.

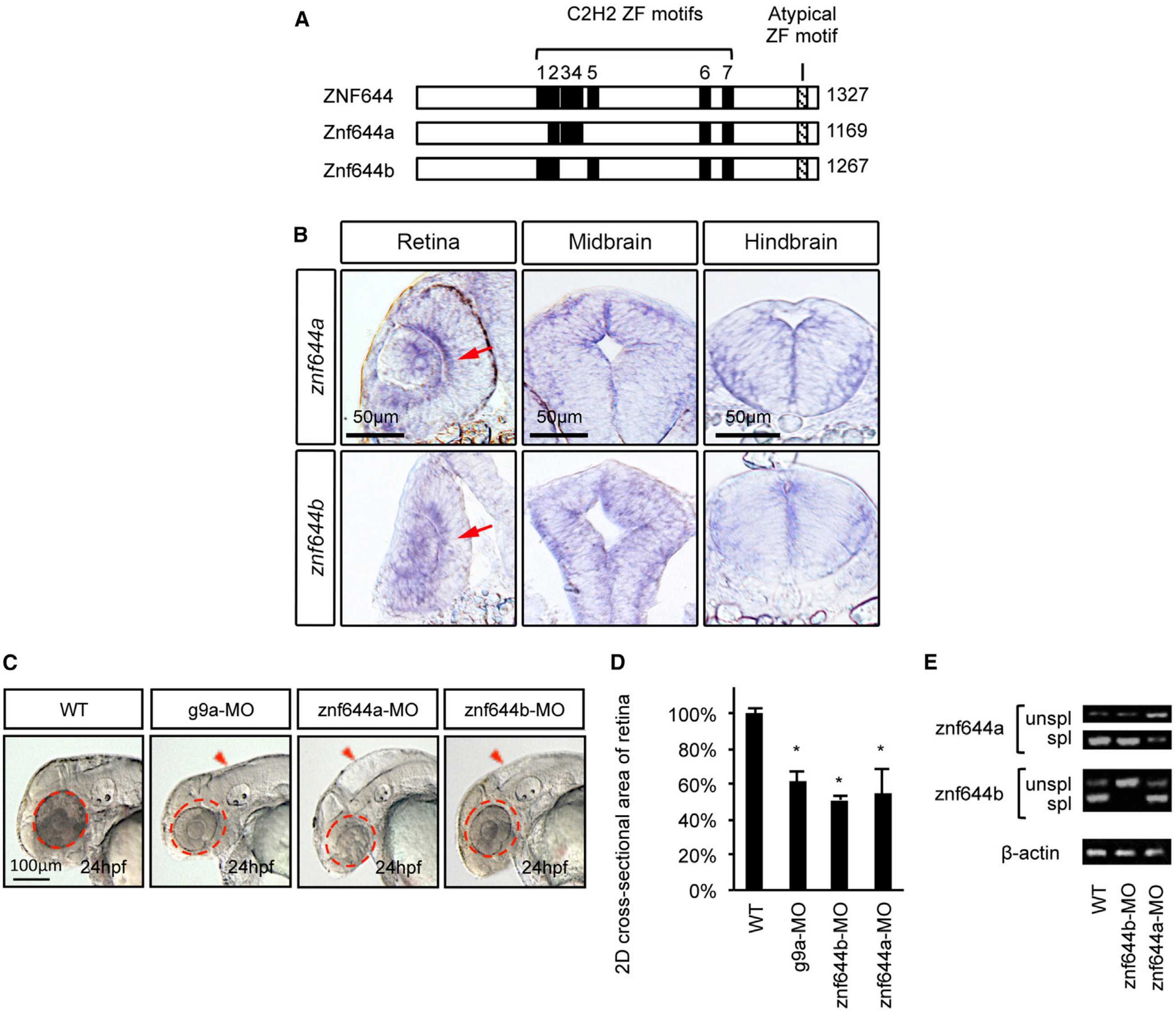

Figure 2. Zebrafish *znf644* Paralogs Are Specifically Expressed In and Regulate the Development of Retinal and Midbrain Progenitor Cells

(A) Conservation of C2H2-like ZF motifs between human and zebrafish ZNF644 genes.

(B) Representative lateral views, retinal cross-sections, and midbrain cross-sections of whole-mount in situ hybridization (WISH) assays monitoring *znf644a* or *znf644b* expression at 24 hpf (n > 15 in each group). Arrows denote the inner part of the central retinal epithelium.

(C) Lateral views of *g9a* (20/32), *znf644a* (10/18), or *znf644b* (14/21) morphant embryos compared with WT (21/21). Dashed circles denote 2D area of the WT retina. Arrowheads denote the dorsal hindbrain region.

(D) Quantitation (mean ± SD) of retinal 2D cross-sectional area (μm^2) of *znf644a*, *znf644b*, or *g9a* morphants relative to WT embryos. Error bars represent SD. *$p < 0.005$, Student's t test.

(E) RT-PCR assays monitoring unspliced (unspl) and spliced (spl) *znf644a* or *znf644b* transcripts at 24 hpf.

G9a/GLP. Indeed, AP-MS assays based on GFP-tagged zebrafish Znf644a or Znf644b proteins in HEK293 cells confirmed the ability to interact with G9a/GLP and WIZ (data not shown). Throughout embryogenesis, *znf644a* and *znf644b* mRNAs were expressed in most tissues, with slightly higher levels in the neuroepithelium of the retina and midbrain at 24 hr post fertilization (hpf) that was reduced by 48 hpf (Figures 2B and S1B–S1D). Since the 24-hpf retina is largely composed of proliferating RPCs on the verge of differentiation (Agathocleous and Harris, 2009; Li et al., 2000), we analyzed the developing retina to investigate potential functional cooperatively with *g9a*.

The Forming Retina and Midbrain Are Disrupted in *znf644a* or *znf644b* Morphants

Loss-of-function studies using g9a-MO were consistent with previous reports (Rai et al., 2010) in that these morphants showed severe morphological abnormalities in a dose-dependent manner (Figures S2A and S2B). Although overall far less severe, splice-block MOs targeted against *znf644a* (znf644a-MO) or *znf644b* (znf644b-MO) showed significant dose-dependent reductions in eye size (microphthalmia) and disrupted midbrain morphology (Figure 2C). Mismatch control MOs showed no discernible phenotypes (data not shown), supporting targeting specificity. MO injection doses eliciting a ~50% reduction in retinal cross-sectional area (Figure 2D) corresponded to a 57% reduction in *znf644a,* nearly complete splicing disruption of *znf644b*, and ~35% drop in *g9a* (Figure 2E and data not shown). Co-injection of cognate mRNA fully rescued the *znf644a* morphant defect and, to a lesser but still appreciable degree, *znf644b* morphants (Figures S2C and S2D), further confirming targeting specificity. These data highlight the developing retina and midbrain as neural structures dependent on coordinate *g9a*/*znf644a*/*znf644b* function.

Znf644a and *znf644b* Regulate Gene Silencing in the Retina via H3K9me2

To assess the extent to which *znf644a* and *znf644b* affect H3K9me2 in vivo, we isolated cells from the head regions (heterogeneous mixture of retinal, brain, and other cell types) of *znf644a* and *znf644b* morphant embryos at 48 hpf and mapped the positioning of H3K9me2 marks by chromatin immunoprecipitation (ChIP) sequencing (GEO: GSE63225). H3K9me2 peaks near transcriptional start sites (TSSs), which were prominent in control embryos, were largely depleted in *znf644a* and *znf644b* morphants (Figures 3A and 3B; Table S1), consistent with roles in the global regulation of H3K9me2.

Although some affected H3K9me2 peaks localized near genes implicated in neural development (Table S1), sample heterogeneity likely precluded sampling of specific loci relevant to neural progenitor cell subpopulations. We therefore performed targeted investigations of the *vsx2* and *ccnd1* genes, two key regulators of multipotency and proliferation reported to be suppressed by G9a/H3K9me2 in mouse RPCs (Katoh et al., 2012). ChIP-PCR assays of zebrafish *vsx2* and *ccnd1* revealed reductions in H3K9me2 in the proximal promoters of *vsx2* and *ccnd1* in *znf644a* and *znf644b* morphant embryos (Figure 3C). In addition, these defects in H3K9me2 coincided with aberrantly mislocalized *vsx2* and *ccnd1* expression throughout the central retina at 48 hpf (Figure 3D), with similar changes observed in *g9a* morphant retinas (Figures S3D and S3E). Collectively, these data demonstrate shared roles for *znf644a* and *znf644b* in H3K9me2-mediated gene silencing in RPCs.

znf644a and *znf644b* Suppress Neural Progenitor Gene Expression and Proliferation

To distinguish whether mislocalized expression of vsx2 and ccnd1 simply represented failed gene silencing during retinal differentiation or a maintained proliferative progenitor identity, we examined additional multipotency (*rx3*, *otx2*) and proneural (*ath5*) markers (Stenkamp, 2007). Strikingly, *znf644a* morphants exhibited elevated and persistent expression of *rx3*, *ath5*, and *otx2* (Figure 3D), consistent with continued expression of multiple genes that typify the RPC expression program. In contrast, *znf644b* morphants showed minimal changes in *rx3* and *ath5* expression, but elevated *otx2* expression (Figure 3D), suggesting the deregulated expression of only a subset of RPC-specific genes. These changes in gene expression were rescued by co-injection of cognate mRNAs (Figures S3A and S3B). Together, these data suggest at least partially non-overlapping requirements for *znf644a* and *znf644b* in suppressing the RPC gene-expression program.

We assessed the proliferative state of retinal cells in the morphants by quantitating 5′-bromo-2-deoxyuridine $(BrdU)^+$ (S phase), phospho-histone H3 $(pH3)^+$ (mitotic), and proliferating cell nuclear antigen $(PCNA)^+$ cells (cycling cells). While the central retina of wild-type (WT) embryos contained few $BrdU^+$ cells (0.23 ± 0.04 cells/μm^2) and low PCNA expression at 48 hpf, there was an order-of-magnitude increase in the number of central $BrdU^+$ cells (1.86 ± 0.29 cells/μm^2) and markedly increased PCNA levels in *znf644a* morphants (Figures 4A and 4B). Likewise, *znf644b* morphants showed a significant (~3.5-fold) increase in $BrdU^+$ cells (0.81 ± 0.28 cells/μm^2) and elevated PCNA (Figures 4A and 4B); however, the number of $pH3^+$ cells was largely unchanged in the *znf644a* or *znf644b* morphant central retina, although a decreasing trend in *znf644a* morphants was noted (Figures 4C and 4D). In contrast, a substantial number of $pH3^+$ cells persisted in the central retina at 72 hpf in *znf644a* morphants, but not *znf644b* morphants, compared with controls, where none were observed (Figures 4C–4E). Likewise, *g9a* morphant retinas exhibited similar increases in the number of proliferating cells that persisted in the central retina into late development (Figures S4A and S4B). Collectively, these results establish roles for *znf644a* and *znf644b* in suppressing retinal cell proliferation.

CRISPR/Cas9 gene editing provided additional confirmation of *znf644b* morpholino target specificity (Figure S5A). Compared with phenotypically normal F1 embryos that were heterozygous for targeted mutations, embryos with two mutant (e.g., frameshift) alleles showed significantly reduced brain and retinal growth and elevated expression of *ccnd1* (Figure S5B), coinciding with the presence of pH3+ cycling cells in the central retina at 48 hpf

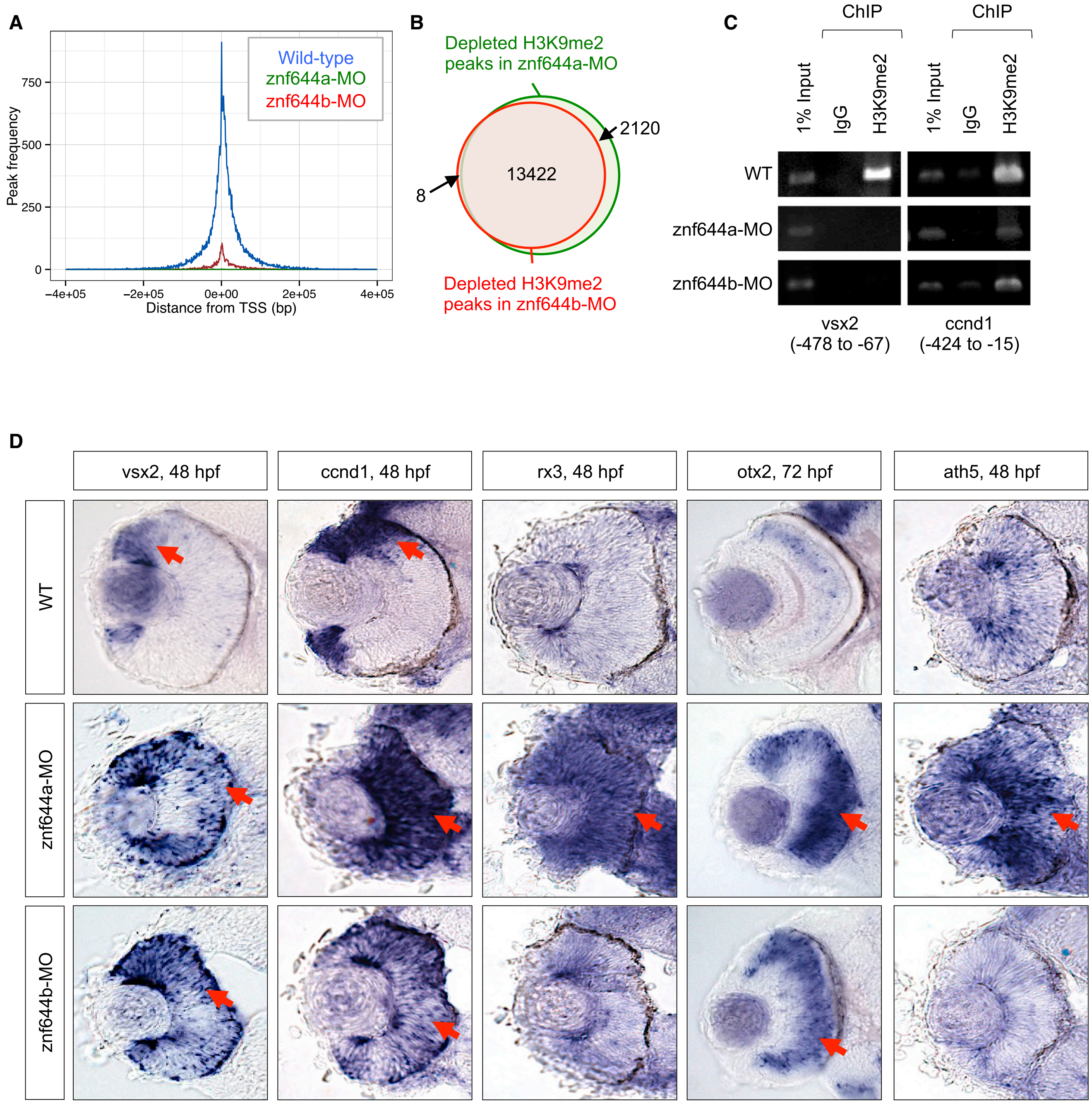

Figure 3. *znf644a* and *znf644b* Are Required for H3K9me2-Mediated Gene Silencing in the Forming Retina

(A) Frequency and distance from TSS at which H3K9me2 peaks were identified in WT, *znf644a*, and *znf644b* morphants.

(B) Venn diagram illustrating the number and overlap of H3K9me2 peaks at 48 hpf in *znf644a* or *znf644b* morphants or WT embryos.

(C) ChIP-PCR assays monitoring H3K9me2 levels near the TSSs of *vsx2* or *ccnd1* genes at 48 hpf.

(D) WISH assays monitoring expression of progenitor or proneural genes in WT, *znf644a*, or *znf644b* morphant retinas. Arrows highlight expression domains.

(Figure S5C). Thus, results from an independent genetic loss-of-function approach are consistent with the phenotype observed in the *znf644b* morphants.

Consistent with the prolonged proliferation and maintained RPC gene-expression program, the central retinas of *znf644a* morphants lacked various types of differentiated

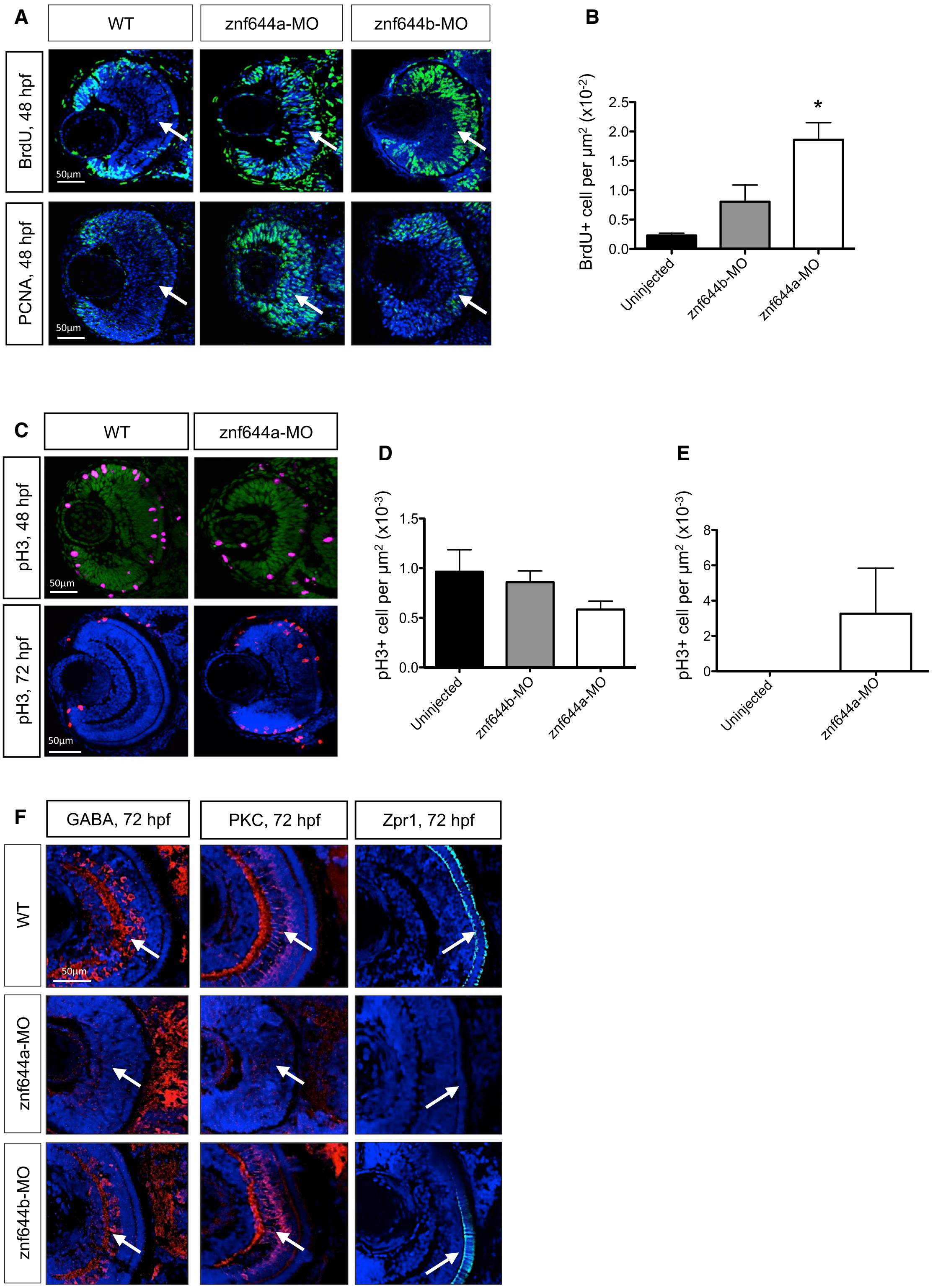

Figure 4. *znf644a* and *znf644b* Morphant Retinas Are Composed of Distinct Populations of Retinal Cells with Differing Characteristics

(A) Immunostaining of retinal cross-sections monitoring BrdU incorporation and PCNA^{+} cells at 48 hpf in WT, *znf644a*, and *znf644b* morphants.

(legend continued on next page)

neurons, as late as 48–72 hpf, including protein kinase C (PKC)$^+$ bipolar neurons, GABA$^+$ amacrine cells, and Zpr1$^+$ cone photoreceptor cells (Figure 4F; data not shown). A similar lack of differentiated retinal neurons was likewise observed in *g9a* morphants (Figure S4C). In contrast, the persistent expression of *vsx2* and *ccnd1* notwithstanding, *znf644b* morphant retinas, as a population, formed differentiated neurons by 48–72 hpf (Figure 4F and data not shown). These data imply distinct regulatory requirements for *znf644a* and *znf644b* vis-à-vis cellular differentiation in the retina.

znf644a Morphant Retinas Preserve Characteristics of Proliferative Progenitors

To further characterize the *znf644a* morphant retina, we assessed the temporal emergence of RGCs and amacrine cells, which are among the first-born differentiated retinal cells (Agathocleous and Harris, 2009; Li et al., 2000). Isl2b$^+$ RGCs and Pax6$^+$ amacrine cells, which are normally amply visible by 48 hpf, were not detected in *znf644a* morphant retinas until ~72 hpf (Figure 5A), consistent with delayed onset of differentiation. Rather, the *znf644a* morphant retinas were also largely composed of Vsx2$^+$ cells at 48 hpf, suggestive of a maintained RPC identity (Figure 5A). We also detected H3K9me2$^+$ nuclei among differentiated cells of the ganglion and inner nuclear layer at 72 hpf (Figure 5A), reflecting global increases in H3K9me2 during differentiation. However, *znf644a* morphant retinas lacked H3K9me2$^+$ nuclei even at 72 hpf (Figure 5A), suggesting that H3K9me2$^+$ nuclei emerge downstream of cell-cycle exit within differentiated retinal neuron populations.

To confirm an RPC-specific role, we transplanted *znf644a* morphant donor cells (membrane-GFP$^+$) into WT host embryos, thereby generating chimeric retinas. We observed a significant (21.6%) increase in pH3$^+$ cells in donor cells (76 cells in total; n = 2) compared with an absence of pH3$^+$ cells in embryos with control donor cells (94 cells in total; n = 2) (Figures S4D and S4E), indicating an RPC-autonomous role of *znf644a*.

Impaired Viability of Differentiated Neurons in *znf644b* Morphant Retinas

Misexpression of proliferation genes during differentiation, as observed in *znf644b* morphants, is known to cause apoptosis (Bonda et al., 2009; Busser et al., 1998; Kuan et al., 2004; Zhu et al., 2004). Indeed, we observed increased cleaved Caspase3 in differentiated regions of *znf644b* morphant retinas at 72 hpf (28.7 ± 10.3) relative to WT retinas (2.0 ± 0.0; Figures 5B and 5C). High levels of apoptotic cells were seen in *g9a* morphant retinas not only at 72 hpf (67.0 ± 10.8), but also at earlier time points (Figures 5B and 5C). Cleaved Caspase3 was rescued in *znf644b* and *g9a* morphants by co-injecting a morpholino targeting p53 (p53-MO), suggesting intrinsic apoptosis pathway activation (Figures 5B and 5C). We also found evidence of concurrent expression of Pax6, a marker for differentiated amacrine and ganglion cells, and PCNA in *znf644b* morphant retinal cells at 56 hpf, coinciding with the onset of excessive cell death (Figure S5E). Overall, these data suggest *znf644b* function is required to maintain the long-term viability of differentiated retinal cells, at least in part through H3K9me2-dependent repression of progenitor and/or cell-cycle genes.

Extensive Functional Cooperativity between *g9a*, *znf644a*, and *znf644b*

We used a genetic cooperativity approach wherein subphenotypic doses of MOs that individually do not yield observable developmental defects were co-injected to reveal potentially additive or synergistic effects. Co-injected subphenotypic g9a-MO and znf644b-MO gave rise to a *g9a* morphant-like phenotype with a substantial number of Caspase3$^+$ apoptotic cells emerging early in the central retina (Figure 6A). Similar cooperativity occurred in mosaic retinas derived from in vivo cell transplantation assays, where a higher incidence (14.6%) of apoptosis was observed among retinal cells derived from co-injected subphenotypic g9a-MO plus znf644b-MO GFP$^+$ donor cells (123 cells in total; n = 4) (Figures S4F–S4H), compared with only 0.56% subphenotypic g9a-MO GFP$^+$ donor cells (142 cells in total; n = 2) or 3.58% subphenotypic znf644b-MO GFP$^+$ donor cells (139 cells in total; n = 3). These data suggest a cell-autonomous role for *g9a* and *znf644b* in maintaining retinal cell viability.

The number of PCNA$^+$ cells localized to the peripheral ciliary marginal zones (CMZ) in control retinas was 64.4 ± 10.2 cells per section (n = 5), with none seen in

(B) Quantitation of BrdU$^+$ cells/μm^2 in central retina of WT, *znf644a*, or *znf644b* morphants at 48 hpf (n = 3 for each group). Error bars represent SD. *$p < 0.05$, Student's t test.
(C–E) Immunostaining of pH3 expression in retinal cross-sections at 48 and 72 hpf in WT, and *znf644a* morphants (C). Quantitation of pH3$^+$ cells/μm^2 in the central retina of WT, *znf644a*, or *znf644b* morphants at (D) 48 hpf (n = 5 for each group) and (E) 72 hpf (n = 2 for each group). Quantitation represented as mean ± SD.
(F) Immunostaining monitoring marker protein expression in retinal cross-sections. At 72 hpf, amacrine cells express GABA, bipolar neurons express PKC, and cone photoreceptors express Zpr1.
White arrows highlight populations of proliferative or differentiated cells.

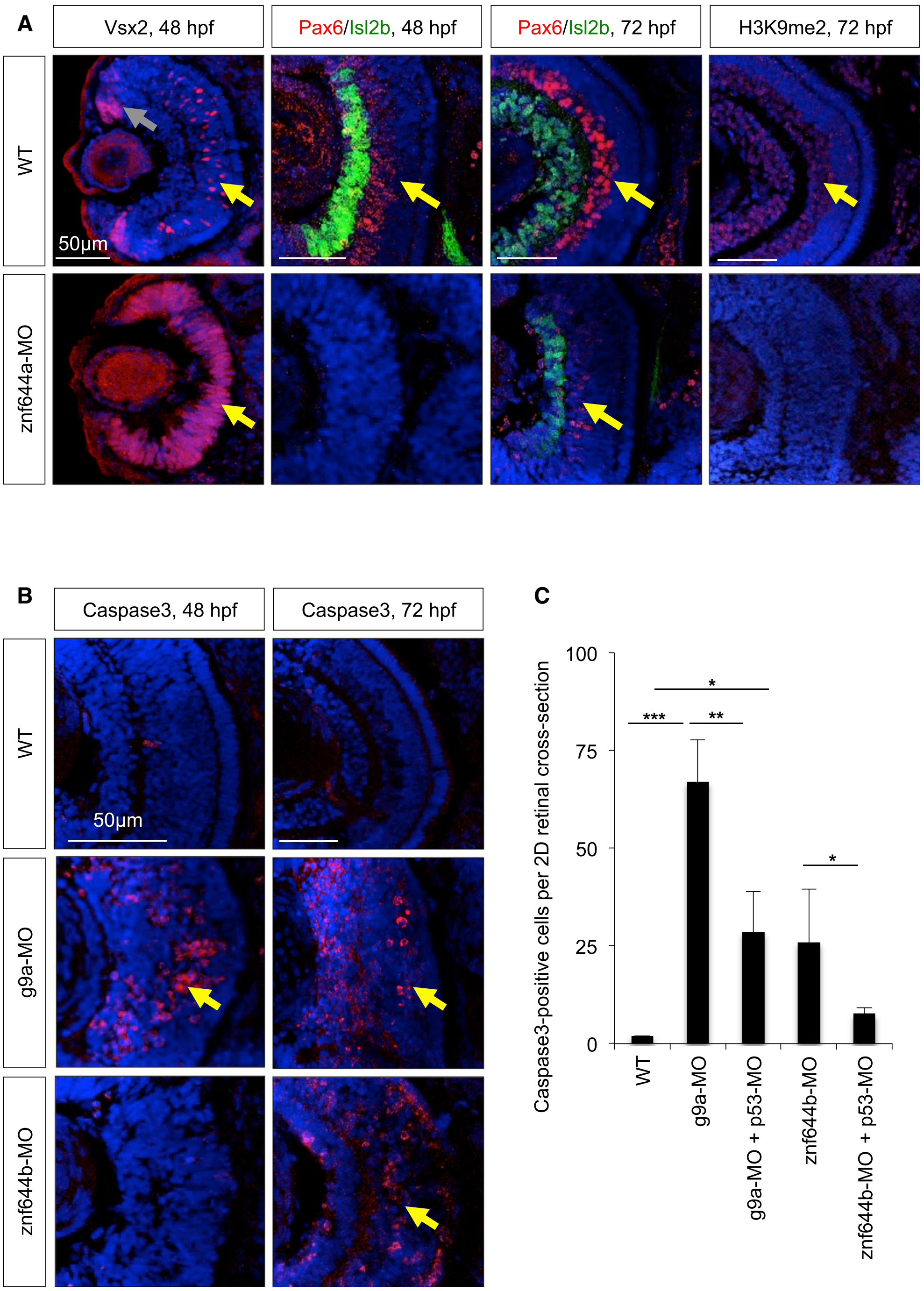

Figure 5. ***znf644a* and *znf644b* Morphant Retinas Exhibit Distinct Cellular Defects**

(A) Immunostaining of retinal cross-sections of Vsx2 (red), Pax6 (red), Isl2b (green), or H3K9me2 (red) in WT or *znf644a* morphant embryos at indicated time points. Vsx2 expression at 48 hpf marks proliferating RPCs in the CMZ (gray arrows) and in a subpopulation of bipolar neurons in the central retina (yellow arrows). Pax6 expression marks amacrine cells and a subset of RGCs, and Isl2b expression marks a subset of RGCs. Differentiated retinal neurons with H3K9me2^{+} nuclei are highlighted.

(B and C) Immunostaining monitoring cleaved Caspase3 (red) in retinal cross-sections from WT, *g9a*, or *znf644b* morphant embryos at 48 or 72 hpf (B). Yellow arrows highlight Caspase3^{+} apoptotic cells. (C) Quantitation of Caspase3^{+} cells in WT, *znf644a* morphant, or *znf644b* morphant retinas with or without co-injection of p53-MO at 72 hpf. Quantitation represented as mean ± SD (n=3 for each group). *$p < 0.05$, **$p < 0.005$, ***$p < 0.0005$, Student's t test.

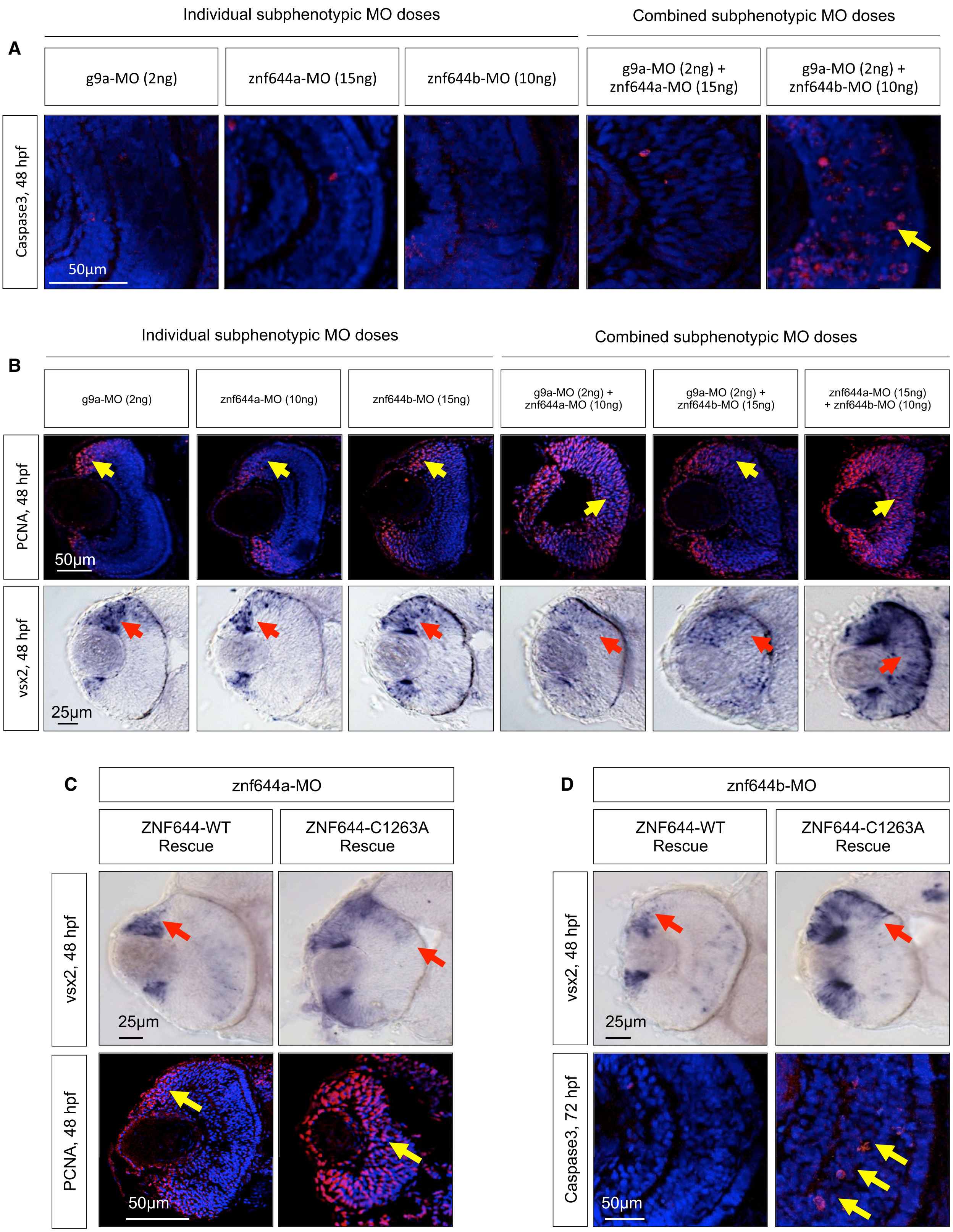

(legend on next page)

the central retina in control or single MO-injected embryos at subphenotypic doses. However, embryos co-injected with subphenotypic doses of znf644b-MO and g9a-MO showed modestly increased PCNA$^+$ cell numbers (74.0 ± 19.7 cells/section; n = 2), but only at the retinal periphery, while no change was observed in the central retina, which correlated with a modest effect in *vsx2* expression (Figure 6B). In contrast, co-injection of znf644a-MO and g9a-MO led to a synergistic increase in PCNA$^+$ cells (129.0 ± 22.6 cells/section; n = 3) throughout the central retina at 48 hpf (Figure 6B), comparable with full *znf644a* morphant retinas (Figure 4A), yet without any observable effects on cell viability (Figure 6A) and with minimal changes in *vsx2* expression (Figure 6B). In addition, compared with controls, where pH3$^+$ cells (17.0 ± 1.0; n = 3) and BrdU$^+$ cells (15.3 ± 6.4; n = 3) were confined primarily to the marginal zone, co-injected subphenotypic doses of g9a-MO and znf644b-MO resulted in marked increased levels in pH3$^+$ (50.6 ± 1.5; n = 3) and BrdU$^+$ cells (31.0 ± 12.5; n = 3) and mislocalized *ccnd1* expression across the entire central retina and margin (Figure S6B). Co-injection of g9a-MO and znf644a-MO resulted in similar overall changes (Figure S6B). These data suggest that extensive functional cooperativity between *g9a* and *znf644b* or *znf644a* maintains the viability of differentiated retinal cells or suppresses a prolonged proliferative state in the retina, respectively.

The misexpression of *vsx2* and *ccnd1* and prolonged retinal cell proliferation in *znf644a* and *znf644b* morphant retinas at 48 hpf (Figures 3A, 4A, and 4B) led us to investigate the role of functional cooperatively among these paralogs in suppressing RPC identity. As seen in *znf644a* and *znf644b* morphants (Figure 3A), co-injection of subphenotypic doses of znf644a-MO plus znf644b-MO resulted in mislocalized *vsx2* and *ccnd1* expression throughout the central retina at 48 hpf (Figures 6B and S6A), consistent with functional cooperativity. In addition, increased numbers of PCNA$^+$ (129.3 ± 25.2 cells/section; n = 6), pH3$^+$ (33.0 ± 6.2; n = 3), and BrdU$^+$ (42.0 ± 9.8; n = 3) cells were evident throughout the central retina (Figures 6B and S6B), suggesting that *znf644a* and *znf644b* work together to suppress RPC gene expression and retinal cell proliferation.

Human ZNF644 Rescues *znf644a* and *znf644b* Morphant Phenotypes

To assess the conservation of human and zebrafish ZNF644 orthologs, we performed functional complementation assays. Full-length native version of human ZNF644 rescued the retinal defects observed in both the *znf644a* and *znf644b* morphant retinas; specifically, mislocalized expression of *vsx2* and elevated apoptosis in *znf644b* morphants (Figures 6D), and the mislocalized expression of *vsx2*, prolonged cellular proliferation, and lack of differentiated neurons in *znf644a* morphants (Figures 6C and S4F). In contrast, the C1263A and H1283A mutants failed to rescue as efficiently (Figures 6C, 6D, S4C, and S4D; data not shown). These data establish functional conservation of human ZNF644 with both zebrafish paralogs and further demonstrate the critical importance of physical association with G9a to ZNF644 function.

Retinal Defects in *znf644a* and *znf644b* Morphants Are Recapitulated in the Midbrain

The cellular and molecular defects of *g9a* and *znf644* morphant retinas were recapitulated in the midbrain, which, unlike other neural regions (Figures S3A and S3C), showed more conspicuous morphological defects. Specifically, *znf644a* and *znf644b* morphant midbrains exhibited upregulated and mislocalized expression of *ccnd1* at 48 hpf

Figure 6. The Retinal Functions of *znf644a* and *znf644b* Are Dependent on Functional and Physical Interactions with *g9a*

(A) Immunostaining of cleaved Caspase3 (red) in retinal cross-sections of embryos injected with individual or combined subphenotypic doses of g9a-MO, znf644a-MO, or znf644b-MO (n = 3 in each group). Yellow arrows highlight apoptotic cells.

(B) (Top) Immunostaining of PCNA (n = 6 in each group) in retinal cross-sections from embryos injected with individual and combined subphenotypic doses of MOs (n = 6 in each group). Yellow arrows denote the position of the PCNA+ population. (Bottom) WISH assays monitoring the expression of *vsx2* in retinal cross-sections of embryos injected with individual subphenotypic doses (n = 10 in each group) and combined subphenotypic co-injection of g9a-MO and znf644a-MO (n = 10), g9a-MO and znf644b-MO (n = 13), and znf644a-MO and znf644b-MO (n = 10). Red arrows denote the position of the vsx2 expressing cells.

(C) (Top) WISH assays in retinal cross-sections monitoring *vsx2* expression at 48 hpf in *znf644a* morphant embryos rescued by co-injection of either WT human ZNF644 mRNA (n = 12) or C1263A mutant mRNA (n = 9). (Bottom) Immunostaining of retinal cross-sections monitoring PCNA expression at 48 hpf showing rescue of *znf644b* morphants by co-injection of WT human ZNF644 mRNA (n = 3) but not C1263A mutants (n = 3).

(D) (Top) WISH assays in retinal cross-sections at 48 hpf monitoring *vsx2* expression from *znf644b* morphant embryos rescued by co-injection of human WT ZNF644 mRNA (n = 14) or C1263A ZNF644 mutant mRNA (n = 9). (Bottom) Immunostaining of retinal cross-sections monitoring cleaved Caspase3 at 72 hpf in *znf644b* morphant embryos rescued by co-injection of WT human ZNF644 mRNA (n = 3) or a C1263A mutant (n = 3). Red arrows highlight *vsx2* expression domains, and yellow arrows highlight PCNA+ cell populations (C) or apoptotic cells (D).

(Figure 7A). Strikingly, combined subphenotypic doses of g9a-MO, znf644a-MO, or znf644b-MO again gave rise to mislocalized *ccnd1* expression in midbrain cells at 48 hpf (Figure S6A), suggesting cooperative roles in gene suppression in the midbrain. As in the retina, *g9a* and *znf644b* morphants exhibited increased apoptosis in the midbrain, which was again rescued by co-injection of p53-MO (Figure 7B). Moreover, *znf644a* morphants exhibited prolonged PCNA expression and elevated numbers of $pH3^{+}$ cells throughout the midbrain (Figure 7C and data not shown). Collectively, these data provide evidence that the cooperative functions of *g9a*, *znf644a*, and *znf644b* in the retina are recapitulated in other populations of neural progenitor cells.

DISCUSSION

ZNF644 Is a Co-regulator of G9a/H3K9me2-Mediated Gene Silencing during Neurogenesis

Our high-confidence interaction dataset recapitulated annotated complexes and many other recently reported protein-protein interactions (Figures 1A and 1B; Table S1). Among the physical interactions identified, we functionally characterized the association of the C2H2-like ZF protein ZNF644, linked to high-grade myopia, with the HMTs G9a and GLP.

As with another documented interactor, WIZ, ZNF644 contains an atypical C2H2-like ZF motif, which we determined is necessary and sufficient for physical association with the G9a and GLP via direct binding to the SET domain (Figures 1D and 1E). These findings contrast with a recent report that a poorly conserved N-terminal region of ZNF644 is responsible for associating with G9a/GLP (Bian et al., 2015) and imply that the conserved atypical C2H2-like ZF motifs of ZNF644 and WIZ constitute a common G9a/GLP recognition module. Point mutations in this atypical C2H2-like ZF motif that abolished interaction with G9a/GLP also failed to rescue *znf644a* and *znf644b* morphant phenotypes (Figures 6C and 6D). Furthermore, using shRNA-based knockdown assays in human cell culture, we determined that ZNF644 specifically co-regulates H3K9me2 levels but not other histone methylation marks, such as H3K9me3 (Figure 1F), suggesting a specialized function for ZNF644 related to G9a/H3K9me2-mediated gene silencing. Collectively, these data establish ZNF644 as a functionally relevant G9a/GLP-interacting protein and a specific co-regulator of H3K9me2 formation.

Multifaceted Regulation of Differentiation by Functional and Physical Interactions

Shortly after 24 hpf, most zebrafish RPCs exit the cell cycle to generate post-mitotic differentiated progeny. These changes in RPC identity occur with concomitant suppression of multipotency-related and cell-cycle-related genes (Dixit et al., 2014), although little is known regarding the physical interactions or tissue-specific co-factors governing this transition. The regulatory properties of ZNF644 vis-à-vis G9a/H3K9me2 and expression patterns of the zebrafish paralogs in neural progenitor cell populations at 24 hpf (but reduced at later stages of retinal development) imply that they are co-regulators of H3K9me2-mediated gene silencing. We showed that disruption of *znf644a* or *znf644b* function leads to downstream defects in retinal differentiation that particularly manifest at 48–72 hpf.

Despite distinct multipotency-related gene-expression programs (e.g., *vsx2* expression in RPCs, but not in midbrain progenitors), similar histone methylation-related complexes may dictate gene silencing and cellular differentiation in distinct populations of neuronal progenitors. While loss of ZNF644 function in cell culture and in vivo leads to deficits in H3K9me2, additional investigations are needed to determine the precise molecular mechanism whereby ZNF644 regulates G9a/GLP activity. Although ZNF644 has multiple C2H2-like ZF motifs and evidence for a role in recruiting G9a/GLP to chromatin has been proposed (Bian et al., 2015), we have failed to establish whether ZNF644 is truly capable of sequence-specific nucleic acid binding (our unpublished data). Neither human nor zebrafish ZNF644 contain the modular three-ZF array typically found in the DNA-binding variety of C2H2 ZF-containing proteins (Lam et al., 2011). One possibility, however, is that ZNF644 may indirectly impart target gene specificity to G9a/GLP by serving as a molecular scaffold bridging HMTs to DNA/RNA-binding repressor molecules and/or other co-repressors to facilitate the stepwise, stable suppression of multipotency- and cell-cycle-related loci. Such a possibility is supported by findings in ESCs that WIZ serves as a physical scaffold bridging G9a/GLP to the CtBP co-repressor complex (Ueda et al., 2006). An alternative possibility, not necessarily mutually exclusive, is that ZNF644 directly stimulates the catalytic activity of G9a/GLP via direct physical interaction to the SET domain. Regardless, given the complex molecular mechanisms involved in G9a/GLP targeting in other contexts (Cedar and Bergman, 2009), additional investigations are needed to define the precise functions of ZNF644 and WIZ in G9a/GLP-mediated silencing during neurogenesis.

Roles for G9a/GLP in regulating cell differentiation in other contexts, such as in HSPCs (Chen et al., 2012) and ESCs (Tachibana et al., 2008), have been reported. In neural progenitors, disruption of G9a/ZNF644 results in the extended maintenance of a proliferative progenitor identity, resulting in delayed formation of differentiated

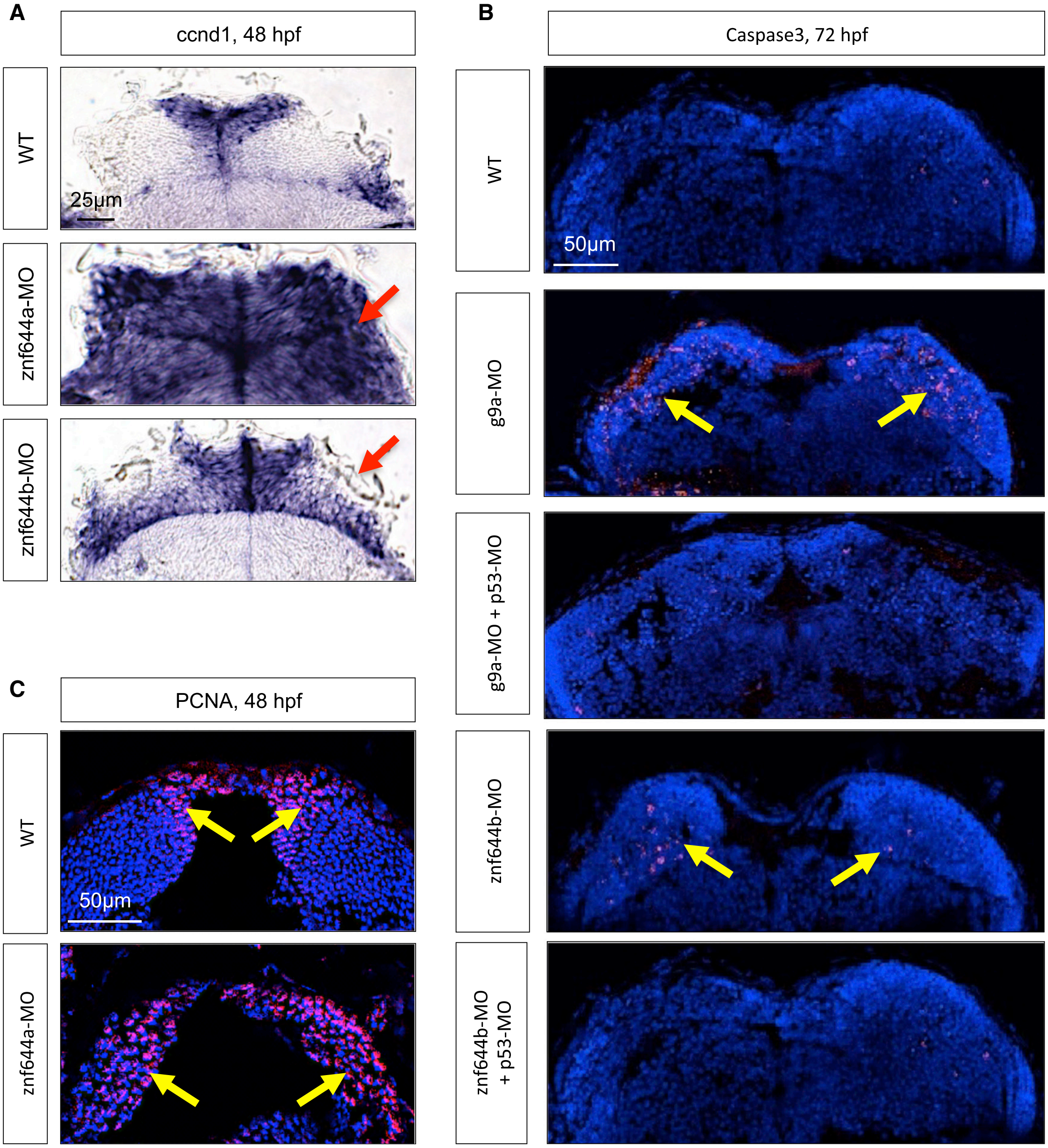

Figure 7. The Retinal Defects of *g9a*, *znf644a*, and *znf644b* Morphants Are Recapitulated in the Midbrain

(A) WISH assays monitoring *ccnd1* expression in WT, *znf644a*, or *znf644b* morphant midbrain cross-sections at 48 hpf. Arrows indicate mislocalized *ccnd1* expression.

(B) Immunostaining of cleaved Caspase3 in *g9a* or *znf644b* morphant midbrain cross-sections at 72 hpf and rescue by p53-MO (n = 3 in each group). Arrows denote the position of Caspase3+ cells.

(C) Immunostaining of PCNA in WT or *znf644a* morphant midbrain cross-sections (n = 3 in each group). Arrows denote the position of PCNA+ cells.

retinal neurons. We observed H3K9me2^{+} nuclei within populations of differentiated retinal neurons, which may represent expanded regions of H3K9me2-marked chromatin reported elsewhere (Wen et al., 2009). The emergence of H3K9me2^{+} nuclei was preceded temporally by suppression of retinal progenitor identity and by expression of lineage commitment, suggesting that formation of expanded H3K9me2-marked chromatin may be a late-phase process occurring within differentiated neuronal cells. Given the reduced retinal expression at 48 or 72 hpf (Figure S1D), when H3K9me2^{+} nuclei are detectable, *znf644a* and *znf644b* are unlikely to directly regulate

formation of expanded regions of H3K9me2-marked chromatin in differentiated cell populations, but rather may regulate initial "seeding" of H3K9me2 marks at isolated genomic regions in retinal cells, at around 24 hpf, such as the promoter regions of *vsx2* and *ccnd1*, as we demonstrated.

Possible Insights into ZNF644 Function and High-Grade Myopia

It is generally thought that longitudinal stretching (or lack thereof) can alter axial elongation, resulting in myopia or hyperopia (Wallman and Winawer, 2004). However, some reports implicate developmental-related genes and signaling pathways in post-natal retinal growth (Hysi et al., 2010; Sundin et al., 2005). In primates, disruption of the CMZ, which harbors a proliferating RPC niche, can cause high-grade myopia (Tkatchenko et al., 2006). It is not obvious how SNPs in the coding sequence and 3′ UTR might affect ZNF644 function. Further investigations are needed to determine the precise role of ZNF644 (and impact of mutations associated with high-grade myopia) in controlling G9a/H3K9me2 during retinal growth and differentiation in adult stem cell populations.

EXPERIMENTAL PROCEDURES

Affinity Purification and Mass Spectrometry

Sequence-verified open reading frames from the Human ORFeome library (Open Biosystems) and UltimateORF collection (Invitrogen) from cDNA were cloned into pDONR221 or pDONR223. Replicate two-step enrichment AP-MS experiments were performed from HEK293 cell lysates essentially as previously described (Mak et al., 2010). GFP-tagged AP-MS assays used Flp-In T-Rex HEK293 cells (a kind gift from Anne-Claude Gingras) cultured in media containing 250 μg/ml hygromycin and induced by treatment with 1 μg/ml doxycycline for 24 hr, followed by affinity purification using 1 μg of rabbit anti-GFP ABfinity recombinant antibody (Invitrogen, G10362). See Supplemental Experimental Procedures for additional information.

Zebrafish Husbandry

Danio rerio (AB strain) were raised and bred at 28°C in accordance with regulations on animal experimentation established by the Canadian Council on Animal Care. Care and procedures were approved by the University of Toronto Animal Care Committee. The *Tg(Isl2b:mGFP)* strain was a kind gift from Dr. Chi-Bin Chien (University of Utah).

Morpholino, mRNA Injections, and Statistics

Embryos were injected at the 1- to 2-cell stage with antisense morpholino oligonucleotides (Gene Tools) and/or in vitro transcribed, capped full-length mRNA. Morpholino sequences, targeted splice junctions, and injection doses are listed in Supplemental Experimental Procedures. RT-PCR assays were performed from cDNA derived from RNA derived from de-yolked embryos (tails removed) using TRIzol (Invitrogen). In all cases, "n" refers to the number of individual embryos assayed in the experiments.

Whole-Mount In Situ Hybridization

Whole-mount in situ hybridization (WISH) was essentially carried out as described in Supplemental Experimental Procedures. RNA antisense probes were obtained from the following sources: *znf644a* and *znf644b*, cloned from 24-hpf embryos; *lef1* and *otx2*, kind gifts from Ashley Bruce; ath5, a kind gift from Jeremy Kay; *rx3*, a kind gift from Pamela Raymond, *vsx2*, Open Biosystems; and *ccnd1* (cb161), ZIRC.

Chromatin Immunoprecipitation

ChIP assays were performed using cells derived from ~100 zebrafish heads at 48 hpf essentially as previously described (Lindeman et al., 2009). In brief, cells were crosslinked using 1% formaldehyde and quenched with 0.125 M glycine. Sonication was performed by Bioruptor (Diagenode) using seven cycles. ChIP was performed using the EZ-ChIP kit (Millipore). H3K9me2 (AbCam, ab1220) and isotype control (AbCam, ab170191) antibodies were used at 2 μg per sample. See Supplemental Experimental Procedures for further information.

Immunostaining and BrdU Incorporation Assays

Samples were fixed in 4% paraformaldehyde and processed as described (Wong et al., 2010). Sections were stained with Hoechst 33,342 and mounted with 80% glycerol. Antibodies used are listed in Supplemental Experimental Procedures. BrdU (5 mM) was injected into the tectal brain ventricle of 48-hpf embryos and fixed with 4% paraformaldehyde, followed by cryosection and immunostaining. Slides were treated with 20 U/mL BrdU, detected using an anti-BrdU antibody (Cedarlane, MCA2060), and imaged by confocal microscopy imaging.

ACCESSION NUMBERS

The accession number for znf644a reported in this paper is RefSeq: NM_001278254.1. The accession number for znf644b reported in this paper is RefSeq: XM_003198781.4.

SUPPLEMENTAL INFORMATION

Supplemental Information includes Supplemental Experimental Procedures, six figures, and four tables and can be found with this article online at http://dx.doi.org/10.1016/j.stemcr.2016.06.012.

AUTHOR CONTRIBUTIONS

J.O. and L.W. designed and conducted most of the experiments, analyzed the data, and wrote the manuscript. S.D, A.M., and H.G. conducted experiments and analyzed data. Y.L. and Z.Z. analyzed data. J.F.G. provided reagents, technical support, and scientific advice. V.T and A.E. designed the study, analyzed the data, wrote the manuscript, and funded the research.

ACKNOWLEDGMENTS

We thank Guoqing Zhong, Xinghua Guo, and Oxana Pogoutse for assistance with affinity purifications as well as Sadhna Phanse for assistance with mass spectrometry data analysis. This work was supported in part by grants from the Ontario Ministry of Innovation (ORF-GL2) and the Canadian Institutes of Health Research (TEAM grant 8294) to A.E.; a grant from the Canadian Institutes of Health Research (MOP-119415) to V.T; an Ontario Graduate Scholarship to J.O; and Vision Science Research Program Scholarships to L.W. and A.M.

Received: November 16, 2015
Revised: June 28, 2016
Accepted: June 29, 2016
Published: August 18, 2016

REFERENCES

Agathocleous, M., and Harris, W.A. (2009). From progenitors to differentiated cells in the vertebrate retina. Annu. Rev. Cell Dev. Biol. *25*, 45–69.

Balemans, M.C., Huibers, M.M., Eikelenboom, N.W., Kuipers, A.J., van Summeren, R.C., Pijpers, M.M., Tachibana, M., Shinkai, Y., van Bokhoven, H., and Van der Zee, C.E. (2010). Reduced exploration, increased anxiety, and altered social behavior: autistic-like features of euchromatin histone methyltransferase 1 heterozygous knockout mice. Behav. Brain Res. *208*, 47–55.

Balemans, M.C., Ansar, M., Oudakker, A.R., van Caam, A.P., Bakker, B., Vitters, E.L., van der Kraan, P.M., de Bruijn, D.R., Janssen, S.M., Kuipers, A.J., et al. (2014). Reduced Euchromatin histone methyltransferase 1 causes developmental delay, hypotonia, and cranial abnormalities associated with increased bone gene expression in Kleefstra syndrome mice. Dev. Biol. *386*, 395–407.

Barton, K.M., and Levine, E.M. (2008). Expression patterns and cell cycle profiles of PCNA, MCM6, cyclin D1, cyclin A2, cyclin B1, and phosphorylated histone H3 in the developing mouse retina. Dev. Dyn. *237*, 672–682.

Bassett, E.A., and Wallace, V.A. (2012). Cell fate determination in the vertebrate retina. Trends Neurosci. *35*, 565–573.

Bian, C., Chen, Q., and Yu, X. (2015). The zinc finger proteins ZNF644 and WIZ regulate the G9a/GLP complex for gene repression. Elife *4*, e05606. http://dx.doi.org/10.7554/eLife.05606.

Bonda, D.J., Evans, T.A., Santocanale, C., Llosa, J.C., Vina, J., Bajic, V., Castellani, R.J., Siedlak, S.L., Perry, G., Smith, M.A., et al. (2009). Evidence for the progression through S-phase in the ectopic cell cycle re-entry of neurons in Alzheimer disease. Aging *1*, 382–388.

Burmeister, M., Novak, J., Liang, M.Y., Basu, S., Ploder, L., Hawes, N.L., Vidgen, D., Hoover, F., Goldman, D., Kalnins, V.I., et al. (1996). Ocular retardation mouse caused by Chx10 homeobox null allele: impaired retinal progenitor proliferation and bipolar cell differentiation. Nat. Genet. *12*, 376–384.

Busser, J., Geldmacher, D.S., and Herrup, K. (1998). Ectopic cell cycle proteins predict the sites of neuronal cell death in Alzheimer's disease brain. J. Neurosci. *18*, 2801–2807.

Cedar, H., and Bergman, Y. (2009). Linking DNA methylation and histone modification: patterns and paradigms. Nat. Rev. Genet. *10*, 295–304.

Cepko, C.L., Austin, C.P., Yang, X., Alexiades, M., and Ezzeddine, D. (1996). Cell fate determination in the vertebrate retina. Proc. Natl. Acad. Sci. USA *93*, 589–595.

Chatr-Aryamontri, A., Breitkreutz, B.J., Oughtred, R., Boucher, L., Heinicke, S., Chen, D., Stark, C., Breitkreutz, A., Kolas, N., O'Donnell, L., et al. (2015). The BioGRID interaction database: 2015 update. Nucleic Acids Res. *43*, D470–D478.

Chen, X., Skutt-Kakaria, K., Davison, J., Ou, Y.L., Choi, E., Malik, P., Loeb, K., Wood, B., Georges, G., Torok-Storb, B., et al. (2012). G9a/GLP-dependent histone H3K9me2 patterning during human hematopoietic stem cell lineage commitment. Genes Dev. *26*, 2499–2511.

Conaway, J.W., Florens, L., Sato, S., Tomomori-Sato, C., Parmely, T.J., Yao, T., Swanson, S.K., Banks, C.A., Washburn, M.P., and Conaway, R.C. (2005). The mammalian Mediator complex. FEBS Lett. *579*, 904–908.

Dixit, R., Tachibana, N., Touahri, Y., Zinyk, D., Logan, C., and Schuurmans, C. (2014). Gene expression is dynamically regulated in retinal progenitor cells prior to and during overt cellular differentiation. Gene Expr. Patterns *14*, 42–54.

Duffy, K.T., McAleer, M.F., Davidson, W.R., Kari, L., Kari, C., Liu, C.G., Farber, S.A., Cheng, K.C., Mest, J.R., Wickstrom, E., et al. (2005). Coordinate control of cell cycle regulatory genes in zebrafish development tested by cyclin D1 knockdown with morpholino phosphorodiamidates and hydroxyprolyl-phosphono peptide nucleic acids. Nucleic Acids Res. *33*, 4914–4921.

Green, E.S., Stubbs, J.L., and Levine, E.M. (2003). Genetic rescue of cell number in a mouse model of microphthalmia: interactions between Chx10 and G1-phase cell cycle regulators. Development *130*, 539–552.

Guttman, M., Donaghey, J., Carey, B.W., Garber, M., Grenier, J.K., Munson, G., Young, G., Lucas, A.B., Ach, R., Bruhn, L., et al. (2011). lincRNAs act in the circuitry controlling pluripotency and differentiation. Nature *477*, 295–300.

Hawthorne, F.A., and Young, T.L. (2013). Genetic contributions to myopic refractive error: Insights from human studies and supporting evidence from animal models. Exp. Eye Res. *114*, 141–149.

Hysi, P.G., Young, T.L., Mackey, D.A., Andrew, T., Fernandez-Medarde, A., Solouki, A.M., Hewitt, A.W., Macgregor, S., Vingerling, J.R., Li, Y.J., et al. (2010). A genome-wide association study for myopia and refractive error identifies a susceptibility locus at 15q25. Nat. Genet. *42*, 902–905.

Katoh, K., Yamazaki, R., Onishi, A., Sanuki, R., and Furukawa, T. (2012). G9a histone methyltransferase activity in retinal progenitors is essential for proper differentiation and survival of mouse retinal cells. J. Neurosci. *32*, 17658–17670.

Kaufmann, I., Martin, G., Friedlein, A., Langen, H., and Keller, W. (2004). Human Fip1 is a subunit of CPSF that binds to U-rich RNA elements and stimulates poly(A) polymerase. EMBO J. *23*, 616–626.

Kleefstra, T., Smidt, M., Banning, M.J., Oudakker, A.R., Van Esch, H., de Brouwer, A.P., Nillesen, W., Sistermans, E.A., Hamel, B.C., de Bruijn, D., et al. (2005). Disruption of the gene Euchromatin Histone Methyl Transferase1 (Eu-HMTase1) is associated with the 9q34 subtelomeric deletion syndrome. J. Med. Genet. *42*, 299–306.

Kuan, C.Y., Schloemer, A.J., Lu, A., Burns, K.A., Weng, W.L., Williams, M.T., Strauss, K.I., Vorhees, C.V., Flavell, R.A., Davis, R.J., et al. (2004). Hypoxia-ischemia induces DNA synthesis without cell proliferation in dying neurons in adult rodent brain. J. Neurosci. *24*, 10763–10772.

Lam, K.N., van Bakel, H., Cote, A.G., van der Ven, A., and Hughes, T.R. (2011). Sequence specificity is obtained from the majority of modular C2H2 zinc-finger arrays. Nucleic Acids Res. *39*, 4680–4690.

Levine, E.M., and Green, E.S. (2004). Cell-intrinsic regulators of proliferation in vertebrate retinal progenitors. Semin. Cell Dev. Biol. *15*, 63–74.

Li, Z., Hu, M., Ochocinska, M.J., Joseph, N.M., and Easter, S.S., Jr. (2000). Modulation of cell proliferation in the embryonic retina of zebrafish (*Danio rerio*). Dev. Dyn. *219*, 391–401.

Lindeman, L.C., Vogt-Kielland, L.T., Alestrom, P., and Collas, P. (2009). Fish'n ChIPs: chromatin immunoprecipitation in the zebrafish embryo. Methods Mol. Biol. *567*, 75–86.

Maier, V.K., Feeney, C.M., Taylor, J.E., Creech, A.L., Qiao, J.W., Szanto, A., Das, P.P., Chevrier, N., Cifuentes-Rojas, C., Orkin, S.H., et al. (2015). Functional proteomic analysis of repressive histone methyltransferase complexes reveals ZNF518B as a G9A regulator. Mol. Cell. Proteomics *14*, 1435–1446.

Mak, A.B., Ni, Z., Hewel, J.A., Chen, G.I., Zhong, G., Karamboulas, K., Blakely, K., Smiley, S., Marcon, E., Roudeva, D., et al. (2010). A lentiviral functional proteomics approach identifies chromatin remodeling complexes important for the induction of pluripotency. Mol. Cell. Proteomics *9*, 811–823.

Marcon, E., Ni, Z., Pu, S., Turinsky, A.L., Trimble, S.S., Olsen, J.B., Silverman-Gavrila, R., Silverman-Gavrila, L., Phanse, S., Guo, H., et al. (2014). Human-chromatin-related protein interactions identify a demethylase complex required for chromosome segregation. Cell Rep. *8*, 297–310.

Margueron, R., and Reinberg, D. (2011). The Polycomb complex PRC2 and its mark in life. Nature *469*, 343–349.

Ni, Z., Olsen, J.B., Guo, X., Zhong, G., Ruan, E.D., Marcon, E., Young, P., Guo, H., Li, J., Moffat, J., et al. (2011). Control of the RNA polymerase II phosphorylation state in promoter regions by CTD interaction domain-containing proteins RPRD1A and RPRD1B. Transcription *2*, 237–242.

Pu, S., Turinsky, A.L., Vlasblom, J., On, T., Xiong, X., Emili, A., Zhang, Z., Greenblatt, J., Parkinson, J., and Wodak, S.J. (2010). Expanding the landscape of chromatin modification (CM)-related functional domains and genes in human. PLoS One *5*, e14122.

Rai, K., Jafri, I.F., Chidester, S., James, S.R., Karpf, A.R., Cairns, B.R., and Jones, D.A. (2010). Dnmt3 and G9a cooperate for tissue-specific development in zebrafish. J. Biol. Chem. *285*, 4110–4121.

Schaefer, A., Sampath, S.C., Intrator, A., Min, A., Gertler, T.S., Surmeier, D.J., Tarakhovsky, A., and Greengard, P. (2009). Control of cognition and adaptive behavior by the GLP/G9a epigenetic suppressor complex. Neuron *64*, 678–691.

Shi, Y., Li, Y., Zhang, D., Zhang, H., Li, Y., Lu, F., Liu, X., He, F., Gong, B., Cai, L., et al. (2011). Exome sequencing identifies ZNF644 mutations in high myopia. PLoS Genet. *7*, e1002084.

Shilatifard, A. (2012). The COMPASS family of histone H3K4 methylases: mechanisms of regulation in development and disease pathogenesis. Annu. Rev. Biochem. *81*, 65–95.

Shinkai, Y., and Tachibana, M. (2011). H3K9 methyltransferase G9a and the related molecule GLP. Genes Dev. *25*, 781–788.

Stenkamp, D.L. (2007). Neurogenesis in the fish retina. Int. Rev. Cytol. *259*, 173–224.

Sundin, O.H., Leppert, G.S., Silva, E.D., Yang, J.M., Dharmaraj, S., Maumenee, I.H., Santos, L.C., Parsa, C.F., Traboulsi, E.I., Broman, K.W., et al. (2005). Extreme hyperopia is the result of null mutations in MFRP, which encodes a Frizzled-related protein. Proc. Natl. Acad. Sci. USA *102*, 9553–9558.

Tachibana, M., Ueda, J., Fukuda, M., Takeda, N., Ohta, T., Iwanari, H., Sakihama, T., Kodama, T., Hamakubo, T., and Shinkai, Y. (2005). Histone methyltransferases G9a and GLP form heteromeric complexes and are both crucial for methylation of euchromatin at H3-K9. Genes Dev. *19*, 815–826.

Tachibana, M., Matsumura, Y., Fukuda, M., Kimura, H., and Shinkai, Y. (2008). G9a/GLP complexes independently mediate H3K9 and DNA methylation to silence transcription. EMBO J. *27*, 2681–2690.

Tkatchenko, A.V., Walsh, P.A., Tkatchenko, T.V., Gustincich, S., and Raviola, E. (2006). Form deprivation modulates retinal neurogenesis in primate experimental myopia. Proc. Natl. Acad. Sci. USA *103*, 4681–4686.

Turner, B., Razick, S., Turinsky, A.L., Vlasblom, J., Crowdy, E.K., Cho, E., Morrison, K., Donaldson, I.M., and Wodak, S.J. (2010). iRefWeb: interactive analysis of consolidated protein interaction data and their supporting evidence. Database (Oxford) *2010*, baq023.

Ueda, J., Tachibana, M., Ikura, T., and Shinkai, Y. (2006). Zinc finger protein Wiz links G9a/GLP histone methyltransferases to the co-repressor molecule CtBP. J. Biol. Chem. *281*, 20120–20128.

Vitorino, M., Jusuf, P.R., Maurus, D., Kimura, Y., Higashijima, S., and Harris, W.A. (2009). Vsx2 in the zebrafish retina: restricted lineages through derepression. Neural Dev. *4*, 14.

Wallman, J., and Winawer, J. (2004). Homeostasis of eye growth and the question of myopia. Neuron *43*, 447–468.

Watanabe, A., Yamada, Y., and Yamanaka, S. (2013). Epigenetic regulation in pluripotent stem cells: a key to breaking the epigenetic barrier. Philosophical transactions of the Royal Society of London Series B. Biol. Sci. *368*, 20120292.

Wen, B., Wu, H., Shinkai, Y., Irizarry, R.A., and Feinberg, A.P. (2009). Large histone H3 lysine 9 dimethylated chromatin blocks

distinguish differentiated from embryonic stem cells. Nat. Genet. *41*, 246–250.

Wong, L., Weadick, C.J., Kuo, C., Chang, B.S., and Tropepe, V. (2010). Duplicate dmbx1 genes regulate progenitor cell cycle and differentiation during zebrafish midbrain and retinal development. BMC Dev. Biol. *10*, 100.

Wong, L., Power, N., Miles, A., and Tropepe, V. (2015). Mutual antagonism of the paired-type homeobox genes, vsx2 and dmbx1, regulates retinal progenitor cell cycle exit upstream of ccnd1 expression. Dev. Biol. *402*, 216–228.

Zhu, X., McShea, A., Harris, P.L., Raina, A.K., Castellani, R.J., Funk, J.O., Shah, S., Atwood, C., Bowen, R., Bowser, R., et al. (2004). Elevated expression of a regulator of the G2/M phase of the cell cycle, neuronal CIP-1-associated regulator of cyclin B, in Alzheimer's disease. J. Neurosci. Res. *75*, 698–703.

Stem Cell Reports, Volume 7

Supplemental Information

G9a and ZNF644 Physically Associate to Suppress Progenitor Gene Expression during Neurogenesis

Jonathan B. Olsen, Loksum Wong, Steven Deimling, Amanda Miles, Hongbo Guo, Yue Li, Zhaolei Zhang, Jack F. Greenblatt, Andrew Emili, and Vincent Tropepe

SUPPLEMENTARY FIGURES AND TABLE LEGENDS

Figure S1. (A) Quantitative (q)PCR assays monitoring the relative expression level of ZNF644 mRNA from HEK293 cell lines stably expressing the indicated shRNA (n=3). Quantitation represented as mean ± SD. A non-silencing shRNA was used as a control. WISH assays monitoring the expression patterns of **(B)** *znf644a* and **(C)** *znf644b*. Both *znf644a* and *znf644b* are expressed maternally, and display widespread expression through the first 24hpf. At 48hpf expression is decreased, but still present in both anterior structures, and throughout the trunk. At each developmental stage no staining is apparent in sense strand control stained embryos. **(D)** RT-PCR assay monitoring the expression of *znf644a*, *znf644b* or β-actin cells from retinal extracts at the indicated time points.

Figure S2: (A) Lateral views of WT or *g9a* morphant embryos illustrating the varying degrees of developmental defects observed. **(B)** Frequency at which the normal/abnormal morphologies are observed in WT or the indicated g9a-MO injected embryos. The mismatch control MO (g9a-mMO) is indicated. **(C)** Lateral views of WT, *znf644a*, or *znf644b* morphant embryos at 48 hpf as well as rescue assays in which the respective cognate mRNA were co-injected. **(D)** The frequency at which the WT or abnormal phenotypes were observed in indicated embryos.

Figure S3. (A) WISH assays monitoring the expression of *vsx2* at 48 hpf or *otx2* at 72 hpf in lateral views or retinal cross-sections from WT, *znf644a* morphant or *znf644b* morphant embryos, as well as morphant embryos rescued by co-injection of the indicated mRNA. Red arrows indicate hindbrain neural cell populations that express *vsx2*. **(B)** Frequency at which the indicated embryos exhibited normal or mislocalized expression

of *vsx2* (top) or *otx2* (bottom). **(C)** WISH assays monitoring the expression of *lef1* at 48 hpf (dorsal views) in WT, *g9a* morphant, *znf644a* morphant, or *znf644b* morphant embryos. Red arrows point to *lef1*-positive midbrain regions, and red arrowheads point to *lef1*-expressing cells of the anterior hypothalamus. **(D)** ChIP-PCR assays monitoring the levels of H3K9me2 at the indicated positions near the TSS of *vsx2* or *ccnd1* genes (as in Figure 3C) at 48 hpf. An anti-H3K9me2 antibody and an isotype control (IgG) antibody were used for ChIP, followed by PCR amplification (35 cycles). Densitometry analysis revealed a ~50% reduction in the levels of H3K9me2 at the *ccnd1* promoter, and complete loss of H3K9me2 levels at the *vsx2* promoter in *g9a* morphant embryos. **(E)** Immunostaining with H3K9me2 antibody demonstrates a global reduction in nuclear expression in the *g9a* morphant retinas at 48 hpf compared to controls.

Figure S4. Top: **(A)** WISH assays monitoring the expression of *vsx2* or *ccnd1* at 48 hpf in WT or *g9a* morphant retinal cross-sections. **(B)** Immunostaining assays monitoring BrdU- or pH3-postive cells at 48 hpf in WT or *g9a* morphant retinal cross-sections. **(C)** Immunostaining assays monitoring the expression of the indicated neuronal markers in *g9a* morphant retinal cross-sections at the indicated time points. Bottom: Blastula cell transplantation experiments (n=3 separate experiments) of donor morphant cells to wildtype host cells. All transplanted embryos were screened at 24 hpf and only those embryos with GFP+ contribution to the retina were further analyzed. **(D-E)** Only 2 out of 30 positively screened transplanted embryos contained GFP+ znf644a-MO cells within the retina at 72 hpf. **(D)** One embryo demonstrated a relatively large population of GFP+ morphant cells near the retinal periphery, which is more permissive for proliferation. Many donor cells at the periphery were also pH3+, and donor morphant cells located at

the central retina were also proliferating ectopically (arrows). **(E)** The other embryo had very few cells in the center retina, but still showed evidence of ectopic proliferation (arrow). In both instances, host central retinal cells were not induced to proliferate. **(F)** Subphenotypic dose of GFP+ g9a-MO in donor cells does not lead to cell death after transplantation. **(G)** Subphenotypic dose of GFP+ znf644b-MO in donor cells does not lead to cell death after transplantation. **(H)** Combined subphenotypic doses of GFP+ g9a-MO and znf644a-MO results in donor cells undergoing cell death (caspase3+) that is confined to the donor cells. In each experiment, n>10 embryos were screened as GFP+ at 24 hpf, and n=3 were sectioned for analysis in each group at 48 hpf. Representative images of sections are shown. The WT *ccnd1* (48 hpf) image in Figure S4A are re-used from Figure 3D; the WT BrdU (48 hpf) in Figure S4B is re-used from Figure 4A; the WT GABA, PKC and Zpr1 (72 hpf) images in Figure S4C are re-used from Figure 4F; and the WT *vsx2* (48 hpf) images in Figure S4C are re-used from Figure 5A.

Figure S5. Top: CRISPR-Cas9 targeting of the *znf644b* gene show multiple mutated alleles in the F_1 generation that correlates with cellular defects in the retina. (**A**) PCR and restriction digest diagnostic on genomic DNA extracted from individual F_0 embryos injected with Cas9 mRNA and sgRNA targeting against exon 2 of *znf644b*. The PCR fragment contains two Bc1I digestion sites, one of which is located within the sgRNA-targeting site. Digestion of wild-type sequences results in three fragments, 449bp, 170bp and 144bp, whereas CRISPR-mediated insertion/deletion mutations result in loss of the digestion site in the target site, resulting in an additional 619bp fragment. (**B,** top) WISH assays monitoring the expression of ccnd1 in F_1 CRISPR embryos at 48hpf. One F1 embryo heterozygous for a frameshift mutation and was phenotypically normal for brain

and retinal growth as well as *ccnd1* expression at 48 hpf (embryo#1). A separate embryo with two mutant (frameshift) alleles showed significantly reduced brain and retinal growth and elevated expression of *ccnd1* (embryo#3), which was reminiscent of the *znf644b* morphant phenotype. (**C,** top) Immunostaining assay monitoring pH3-positive cells in retinal cross-sections from F_1 CRISPR embryos at 48hpf. One embryo had two *znf644b* mutant alleles, one of which was predicted to be a very large deletion likely resulting in a non-functional protein, while the other allele had a missense mutation leading to a predicted substitution of only two amino acids. The overall phenotype of this embryo was comparable to WT, suggesting that this embryo was functionally heterozygous (embryo#1). Other embryos genotyped as heteroallelic mutants displayed phenotypes that were similar to the morphants, such as a reduced retinal size and persistent pH3+ cells in the central retina at 48 hpf (embryo#2: 11.67 ± 4.16; embryo #3: 12.67 ± 2.08 compared to WT: 3.8 ± 0.5, n=9). (**B** & **C,** bottom) The corresponding sequencing data for both alleles of the embryos analyzed, with the predicted effect on the protein function. Black letters represent unchanged bases; Blue letters represent the PAM sequence; Red letters represent the target sequence; Pink letters represent the Bc1I digestion site; Dashes represent deletions; Green letters represent inserted bases. Bottom: Expression overlap of cell cycle regulator PCNA and the retinal differentiation marker Pax6 in the *znf644b* morphant retina. At 56 hpf, which coincides with the onset of widespread cell death of central retinal cells in *znf644b* morphants, PCNA can be found to be expressed in differentiated amacrine and ganglion cells (Pax6-postive) in the *znf644b* morphant retina, even though this population of neurons is reduced overall.

Figure S6. Interaction of *g9a* and *znf644* genes in regulating progenitor cell cycle. **(A)** WISH assays monitoring the expression of *ccnd1* in (top) midbrain or (bottom) retinal cross-sections at 48 hpf from WT embryos or embryos injected with the indicated combined subthreshold MO doses. **(B)** Immunostaining assays monitoring pH3 levels or BrdU incorporation at 48 hpf from WT or embryos injected with the indicated combined subthreshold MO doses. **(C)** Rescue experiments in which mRNA encoding WT human (ZNF644) or mutant version lacking G9a/GLP binding capability (C1263A) is co-injected with znf644a-MO (left) or znf644b-MO (right). *Vsx2* expression was assessed by WISH at 48 hpf, and PCNA, pH3, and Zpr1 levels (n=3 in each group) were assessed by immunostaining and confocal microscopy at the indicated time points.

Table S1. LC-MS/MS dataset for 33 replicate AP-MS experiments for histone methylation- and transcription-related proteins. Bait-prey interactions are indicated, as are the interaction scores based on a SAINT cut-off of ≥ 0.83.

Table S2. Dataset for replicate AP-MS assays using GFP-tagged ZNF644. Bait proteins and replicate number are indicated in column headers, with identified prey proteins as row headers. Numerical values represent spectral counts based on LC-MS/MS data.

Tables S3-S4. WT vs znf644a-MO (S3) and WT vs znf644b-MO. Positioning of H3K9me2 peaks from ChIP-seq in znf644a-MO or znf644b-MO relative WT embryos at 48 hpf.

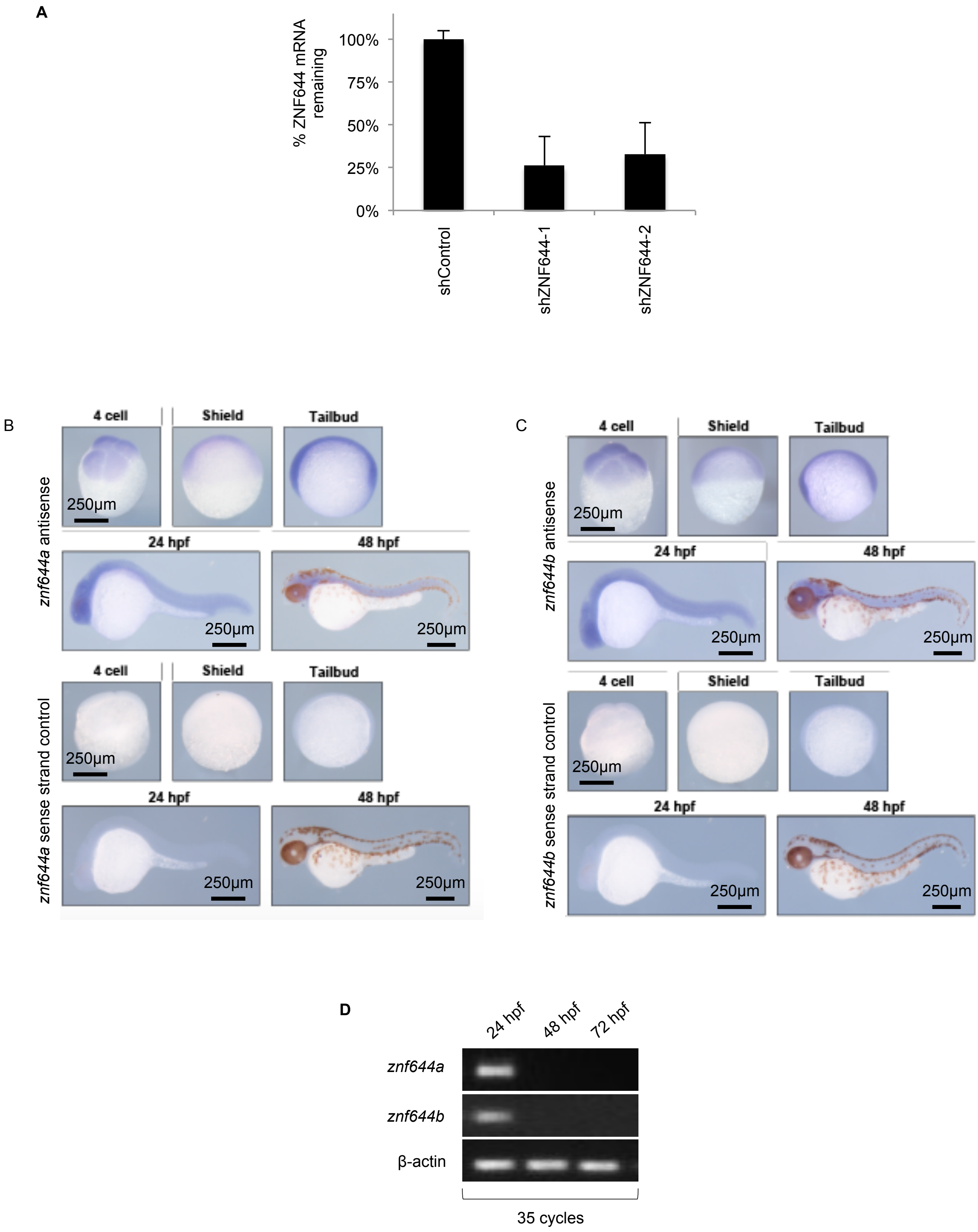

A
% ZNF644 mRNA remaining
100%
75%
50%
25%
0%
shControl
shZNF644-1
shZNF644-2
B
4 cell
Shield
Tailbud
znf644a antisense
250μm
24 hpf
48 hpf
znf644a sense strand control
C
znf644b antisense
znf644b sense strand control
D
24 hpf
48 hpf
72 hpf
znf644a
znf644b
β-actin
35 cycles

Figure S1

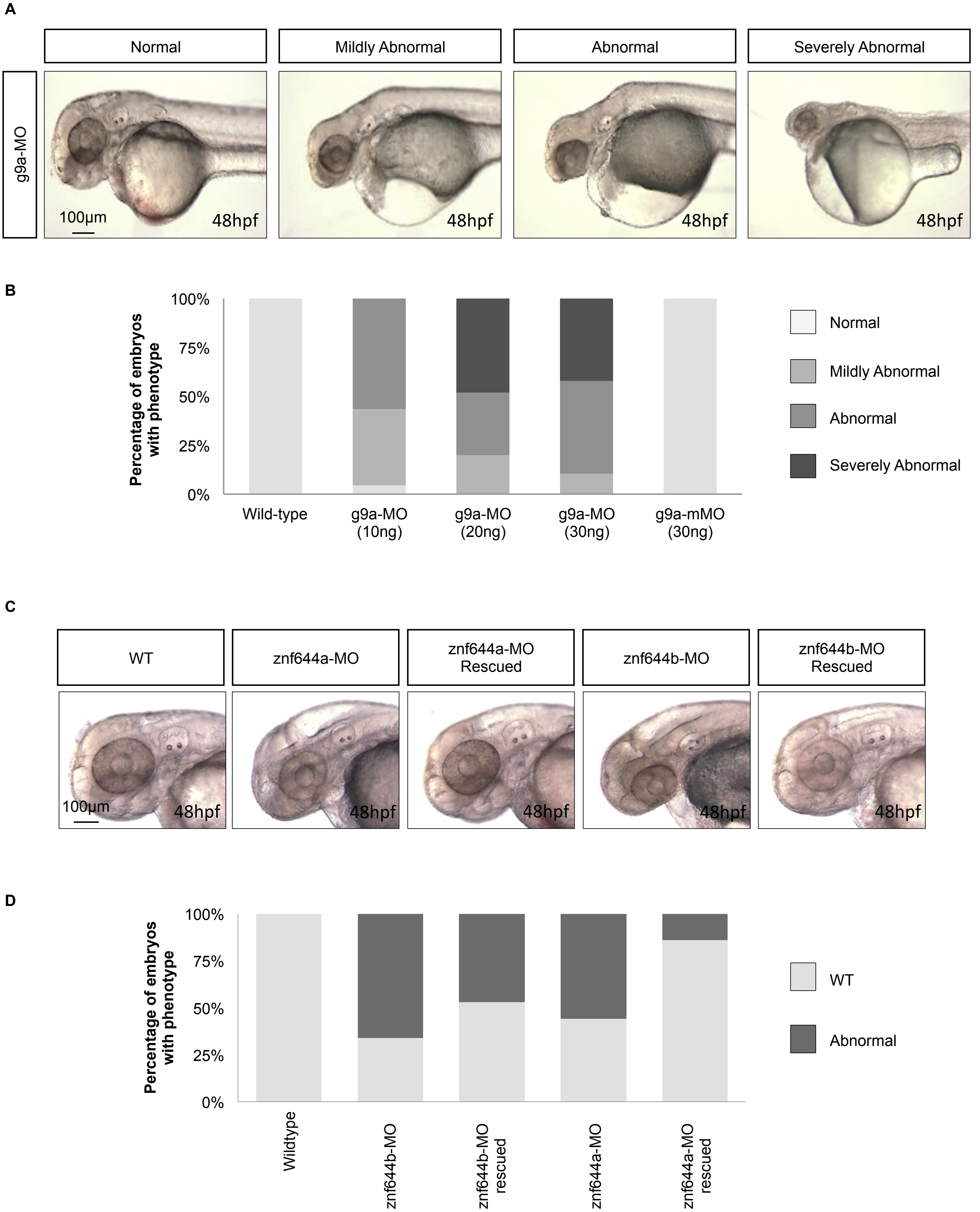
A
Normal
Mildly Abnormal
Abnormal
Severely Abnormal
g9a-MO
100μm
48hpf
B
Percentage of embryos with phenotype
100%
75%
50%
25%
0%
Wild-type
g9a-MO (10ng)
g9a-MO (20ng)
g9a-MO (30ng)
g9a-mMO (30ng)
Normal
Mildly Abnormal
Abnormal
Severely Abnormal
C
WT
znf644a-MO
znf644a-MO Rescued
znf644b-MO
znf644b-MO Rescued
100μm
48hpf
D
Percentage of embryos with phenotype
100%
75%
50%
25%
0%
Wildtype
znf644b-MO
znf644b-MO rescued
znf644a-MO
znf644a-MO rescued
WT
Abnormal

Figure S2

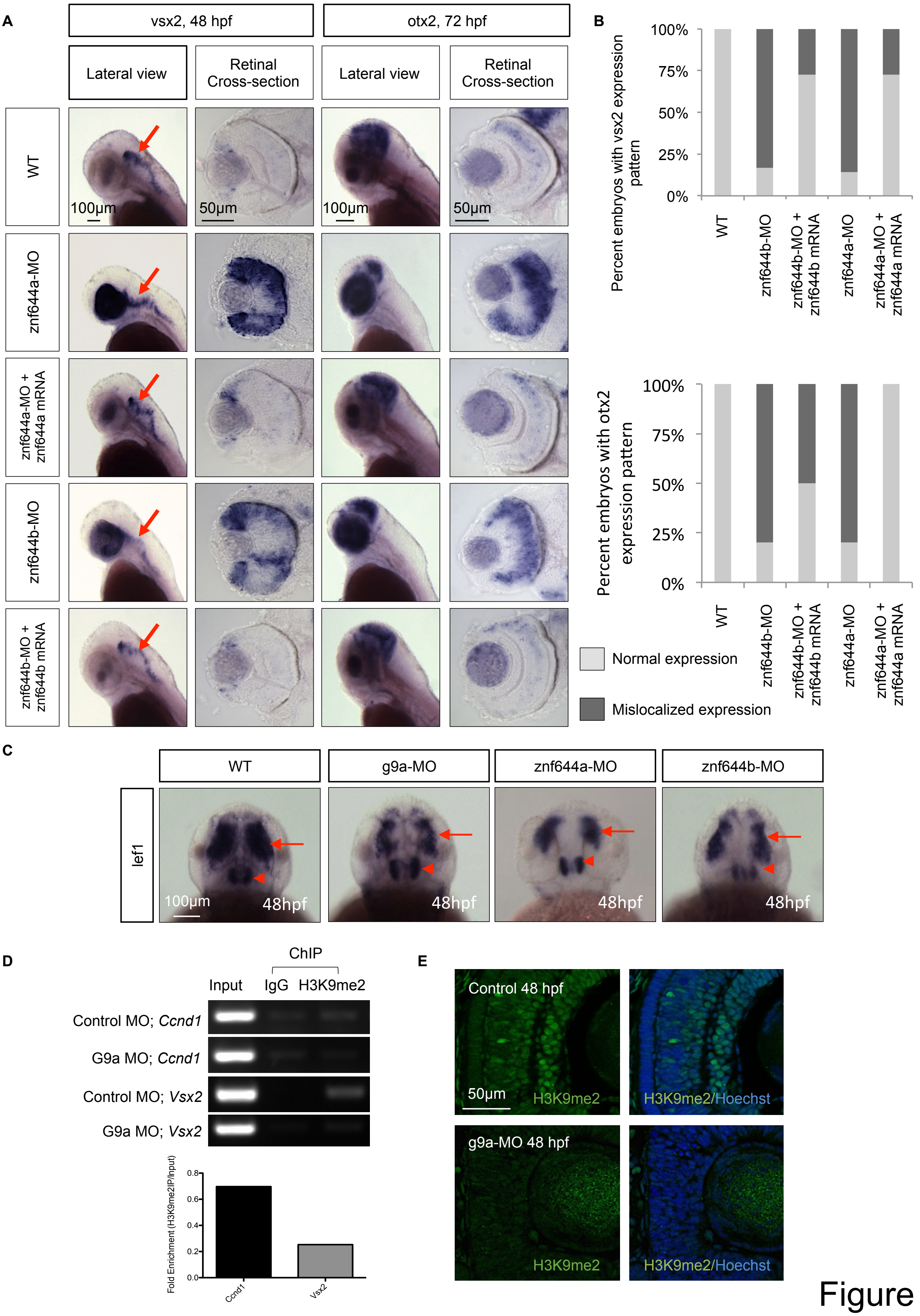
A
vsx2, 48 hpf
otx2, 72 hpf
Lateral view
Retinal Cross-section
Lateral view
Retinal Cross-section
WT
znf644a-MO
znf644a-MO + znf644a mRNA
znf644b-MO
znf644b-MO + znf644b mRNA
100µm
50µm
100µm
50µm
B
Percent embryos with vsx2 expression pattern
100%
75%
50%
25%
0%
WT
znf644b-MO
znf644b-MO + znf644b mRNA
znf644a-MO
znf644a-MO + znf644a mRNA
Percent embryos with otx2 expression pattern
100%
75%
50%
25%
0%
WT
znf644b-MO
znf644b-MO + znf644b mRNA
znf644a-MO
znf644a-MO + znf644a mRNA
Normal expression
Mislocalized expression
C
WT
g9a-MO
znf644a-MO
znf644b-MO
lef1
100µm
48hpf
48hpf
48hpf
48hpf
D
ChIP
Input
IgG
H3K9me2
Control MO; Ccnd1
G9a MO; Ccnd1
Control MO; Vsx2
G9a MO; Vsx2
Fold Enrichment (H3K9me2IP/Input)
0.8
0.6
0.4
0.2
0.0
Ccnd1
Vsx2
E
Control 48 hpf
50µm
H3K9me2
H3K9me2/Hoechst
g9a-MO 48 hpf
H3K9me2
H3K9me2/Hoechst

Figure S3

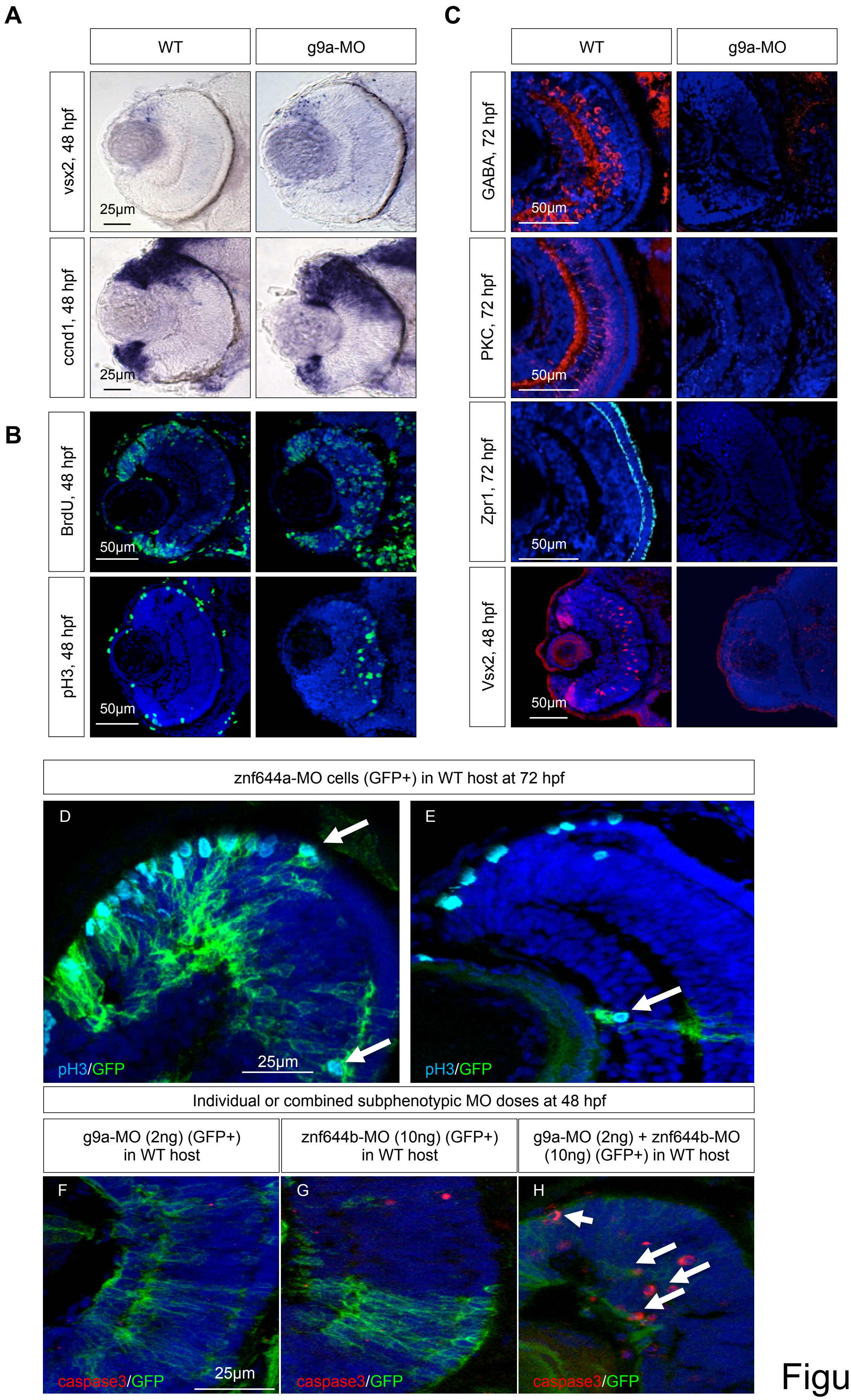
A
WT
g9a-MO
vsx2, 48 hpf
25µm
ccnd1, 48 hpf
25µm
B
BrdU, 48 hpf
50µm
pH3, 48 hpf
50µm
C
WT
g9a-MO
GABA, 72 hpf
50µm
PKC, 72 hpf
50µm
Zpr1, 72 hpf
50µm
Vsx2, 48 hpf
50µm
znf644a-MO cells (GFP+) in WT host at 72 hpf
D
pH3/GFP
25µm
E
pH3/GFP
Individual or combined subphenotypic MO doses at 48 hpf
g9a-MO (2ng) (GFP+) in WT host
znf644b-MO (10ng) (GFP+) in WT host
g9a-MO (2ng) + znf644b-MO (10ng) (GFP+) in WT host
F
caspase3/GFP
25µm
G
caspase3/GFP
H
caspase3/GFP

Figure S4

A ***Znf644b*** **target (exon 2)**

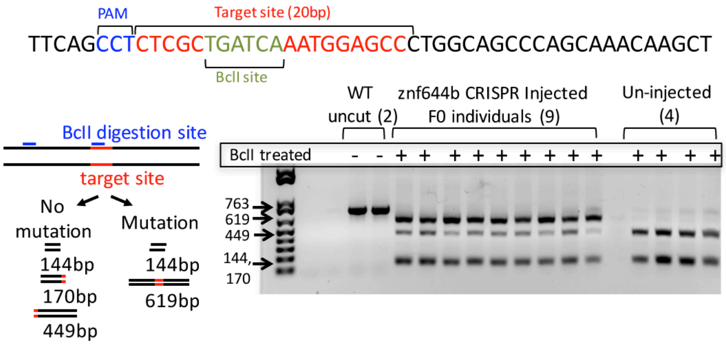

B

znf644b CRISPR F_1 embryos

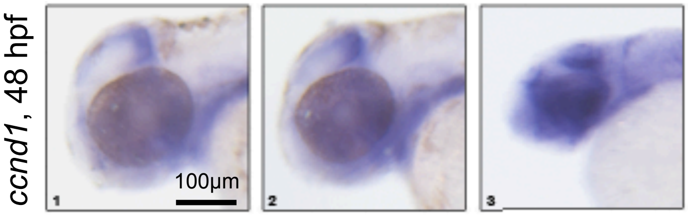

	INDEL	EFFECT ON PROTEIN
WT Allele: TTCAGCCTCTCGCTGATCAAATGGAGCCCTGGCAGCCCAGCAAACAAGCT		
1-Allele 1: TTCAGCCTCTCGCTGATCAAATGGAGCCCTGGCAGCCCAGCAAACAAGCT	0	NO EFFECT
1-Allele 2: TTCAGC - - - - - - - TGATCAAATGGAGCCCTGGCAGCCCAGCAAACAAGCT	-7	FRAMESHIFT
2-Allele 1: TTCAGCCTCTC - - - - - - - AATGGAGCCCTGGCAGCCCAGCAAACAAGCT	-7	FRAMESHIFT
2-Allele 2: TTCAGCC - - - - - GT - - - - - - AAATGGAGCCCTGGCAGCCCAGCAAACAAGCT	-11, +2	MISSENSE MUTATION
3-Allele 1: TTCAGCCTCTC - - - - - TCAAATGGAGCCCTGGCAGCCCAGCAAACAAGCT	-5	FRAMESHIFT
3-Allele 2: TTCAGCCTCT - - - - GATCAAATGGAGCCCTGGCAGCCCAGCAAACAAGCT	-4	FRAMESHIFT

C

znf644b CRISPR F_1 embryos

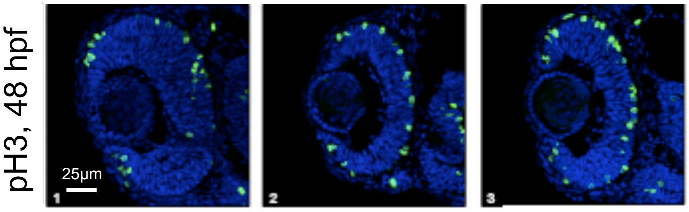

	INDEL	EFFECT ON PROTEIN
WT Allele: TTCAGCCTCTCGCTGATCAAATGGAGCCCTGGCAGCCCAGCAAACAAGCT		
1-Allele 1: TTCAGCCTGAAATGGATCAAATGGAGCCCTGGCAGCCCAGCAAACAAGCT	-6, +6	MISSENSE
1-Allele 2: UNKNOWN LARGE DELETION	>310	LARGE DELETION
2-Allele 1: TTCAGCCTCTCCAAAGCTGATCAAATGGAGCCCTGGCAGCCCAGCAAACAAGCT	+4	FRAMESHIFT
2-Allele 2: TTCAGCCTCTC - - - - - - - TCGGATGC - - - - - - AAATGGAGCCCTGGCAGCCCAGCAA	-13, +8	FRAMESHIFT
3-Allele 1: TTCAGCCTCT - - - - - ATCAAATGGAGCCCTGGCAGCCCAGCAAACAAGCT	-5	FRAMESHIFT
3-Allele 2: TTCAGCCT - - - - GCCGGAGCC - - - - TCAAATGGAGCCCTGGCAGCCCAGCAAACAA	-8, +9	FRAMESHIFT

E

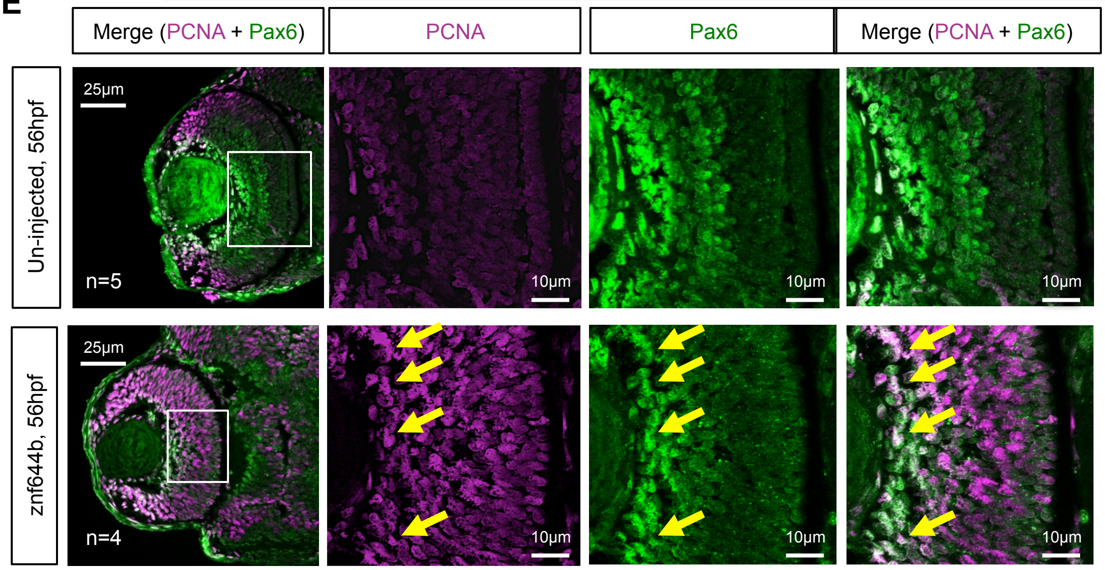

Figure S5

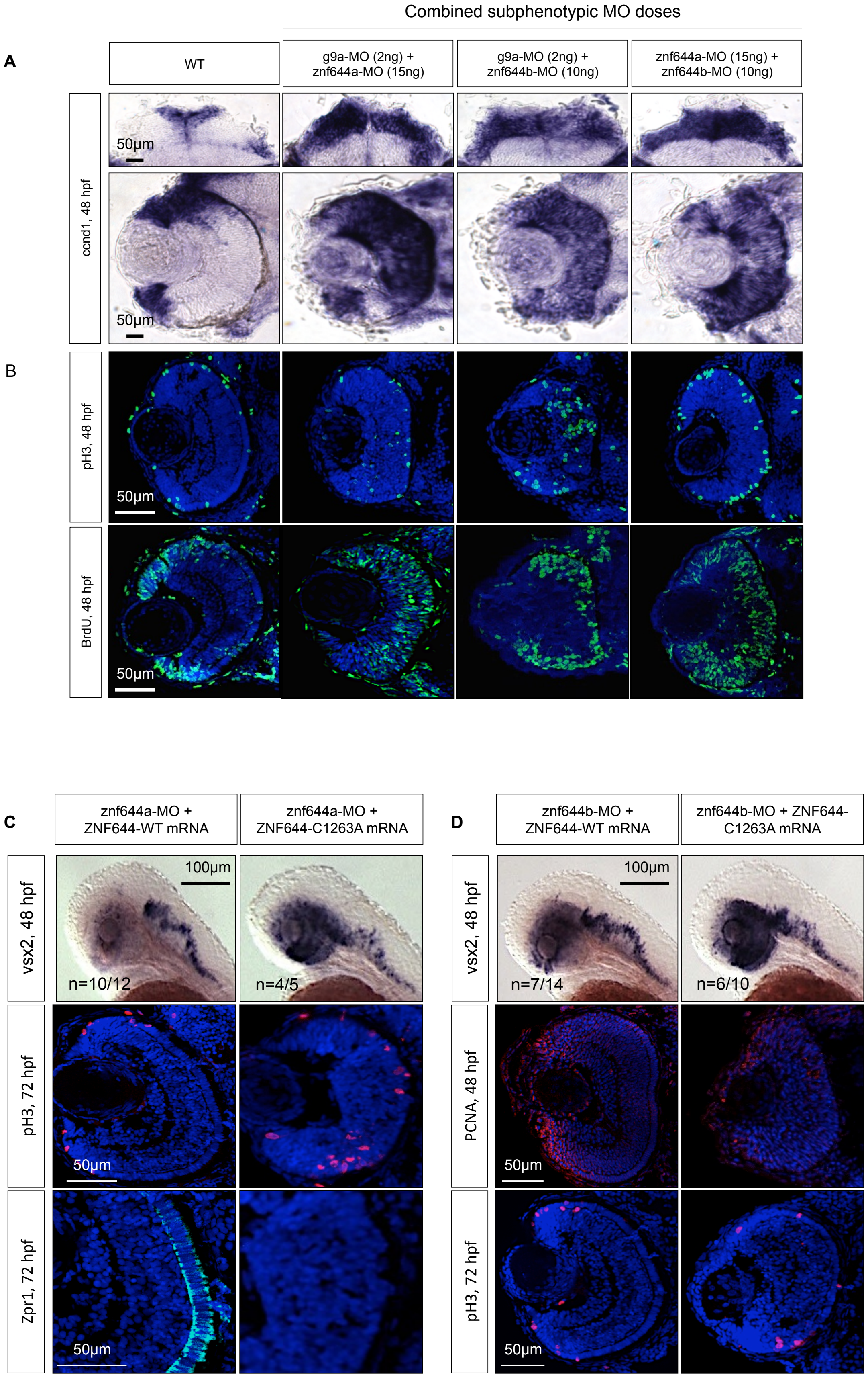
Combined subphenotypic MO doses
A
WT
g9a-MO (2ng) + znf644a-MO (15ng)
g9a-MO (2ng) + znf644b-MO (10ng)
znf644a-MO (15ng) + znf644b-MO (10ng)
ccnd1, 48 hpf
50µm
50µm
B
pH3, 48 hpf
50µm
BrdU, 48 hpf
50µm
C
znf644a-MO + ZNF644-WT mRNA
znf644a-MO + ZNF644-C1263A mRNA
vsx2, 48 hpf
100µm
n=10/12
n=4/5
pH3, 72 hpf
50µm
Zpr1, 72 hpf
50µm
D
znf644b-MO + ZNF644-WT mRNA
znf644b-MO + ZNF644-C1263A mRNA
vsx2, 48 hpf
100µm
n=7/14
n=6/10
PCNA, 48 hpf
50µm
pH3, 72 hpf
50µm

Figure S6

SUPPLEMENTAL METHODS

RNA interference and western blotting

HEK293 cells stably-expressing shRNA targeting Luciferase (non-silencing control), G9a or ZNF644 were generated as previously described (Mak et al., 2010). shRNA clones (kind gifts from Jason Moffat) are as follows: shControl (TRCN0000072256), shG9a-1 (TRCN0000115667), shG9a-2 (TRCN0000115668), shZNF644-1 (TRCN0000140402), and shZNF644-2 (TRCN0000145205). The following antibodies were used for immunodetection: rabbit anti-G9a (C6H3, Cell Signalling, #3306), rabbit anti-H3K9me2 (D85B4, Cell Signalling, #4658), rabbit anti-H3K9me3 (AbCam, ab8898), mouse anti-GAPDH (6C5, AbCam, ab8245), and rabbit anti-H3 antibody (AbCam, ab1791). Validation of shRNA-mediated knockdown of ZNF644 was determined by qPCR and quantified by the $\Delta\Delta$Ct method.

Morpholino injections

Morpholinos doses used in this study are as follows: g9a-MO and control MO (Rai et al., 2010), 4ng; znf644a-MO (ATTAAAATTGTCACCTGTTTTGACT) and control MO (ATTTAATTTGTCAGCTGTATTCACT), 20ng; and znf644b-MO (GTGAGCAATAATCACCTTTTCTGAT) and control MO (GTGTGCATAATCTCCTTATCAGAT), 30ng. p53-MO was used as previously described (Robu et al., 2007).

Whole-mount in situ hybridization (WISH)

WISH was carried out as previously described (Deimling et al., 2015) with the following modifications: Embryos were digested in 5µg/ml Proteinase K and re-fixed for 20 min in 4% paraformaldehyde; TTw with 2mg/ml BSA was used in place of MAB on days 2 and 3, and

embryos were incubated in TTw-BSA overnight on day 3 before staining on day 4. Samples were washed out of glycerol and into sucrose gradients for cryosectioning as described (Wong et al., 2010).

ChIP-PCR and ChIP-seq analysis

ChIP-PCR primers were as follows (5'→3'): vsx2-F, AGACATTTTCCAGCGCACTT; vsx2-R, GCAATACGCATGATCCCTCT; ccnd1-F, TTCACCCCAGTCTTTTCCAC; and ccnd1-R, AAAGTCTCGCTGCAGCTCTC-3'. Alignments were performed by Bowtie2 (Langmead and Salzberg, 2012) and peak calling by MACS2 (Feng et al., 2012). Promoter regions of *vsx2* and *ccnd1* were amplified by PCR (35 cycles).

Immunostaining and BrdU incorporation assays

The following antibodies were used at the indicated dilutions: Caspase3 (cleaved, Asp175; Cell Signaling Technology, #9961), H3K9me2 (AbCam, ab1220) Pax6a (Covance, PRB-278P), PCNA (ZYMED Laboratories, 13-3900), pH3 (D2V8, Cell Signaling Technology, #3377), PKC (Santa Cruz, sc-209), Zn5 (ZIRC), Zpr1 (ZIRC), GABA (Sigma-Aldrich), anti-rabbit Cy3 (Jackson ImmunoResearch Laboratories, #111-165-003), anti-mouse Cy3 (Jackson ImmunoResearch Laboratories, #115-165-146), anti-mouse Cy5 (Jackson ImmunoResearch Laboratories, #115-165-146), and Vsx2 (a kind gift from Shin-ichi Higashijima).

Blastula transplantations

Donor cells from *Tg(β-actin:mGFP)* embryos injected with g9a-MO, znf644a-MO or znf644b-MO were transplanted into AB strain host embryos between 3-4 hpf. Only hosts with GFP+ retinal cells were cryosectioned for analysis using immunostaining.

CRISPR-Cas9 Mutagenesis

Targeting sites for CRISPR-Cas9 mediated mutagenesis were designed against znf644b using CHOPCHOP (https://chopchop.rc.fas.harvard.edu/index.php). The target site used for znf644b was GGCTCCATTTGATCAGCGAG. The single guide RNA (sgRNA) was transcribed directly from long oligonucleotides containing a T7 promoter, targeting sequence, and guide RNA sequence using MEGAshortscript (Ambion AM1354). The sgRNA was purified using ethanol/ammonium acetate precipitation. Cas9 mRNA was transcribed from XbaI-linearized pT3TS-nCas9n plasmid using T3 MEGAscript kit (Ambion AM1338) and purified using lithium chloride precipitation. sgRNA (800pg) and Cas9 (600pg) mRNA were co-injected into the cell of 1-cell staged zebrafish embryos. Genomic DNA from a portion of injected embryos was individually extracted and a region around the target site was PCR amplified (F primer TGTGCCAAGACTGAAGACTGTT; R primer AGTCATCTCCCTCTTGTGGTGT), purified and digested with the restriction enzyme Bc1I (NEB) to assess for mutations. The remaining embryos were raised to adulthood. Once adulthood was reached, the fish were in-crossed and similarly assessed for germ-line transmitted mutations in the F_1 progeny via the restriction digest diagnostic. The DNA extracted from individual F_1 embryos was also sequenced to confirm the extent and type of mutation. To correlate genotype to phenotype the gDNA was extracted from the tail of the embryos, PCR and sequenced, while the head was used for in situ hybridization and immunohistochemistry.

MASS SPECTROMETRY AND DATA ANALYSIS

After identification of interacting proteins using a precision hybrid Orbitrap-Velos mass spectrometer (ThermoFisher Scientific)(Walkey et al., 2012), confidence values were assigned to

protein-protein interactions using a strict Statistical Analysis of Interactome (Choi et al., 2011) algorithm cut-off score of ≥0.83.

REFERENCES

Choi, H., Larsen, B., Lin, Z.Y., Breitkreutz, A., Mellacheruvu, D., Fermin, D., Qin, Z.S., Tyers, M., Gingras, A.C., and Nesvizhskii, A.I. (2011). SAINT: probabilistic scoring of affinity purification-mass spectrometry data. Nature methods *8*, 70-73.

Deimling, S.J., Halabi, R.R., Grover, S.A., Wang, J.H., and Drysdale, T.A. (2015). Understanding early organogenesis using a simplified in situ hybridization protocol in Xenopus. Journal of visualized experiments : JoVE, e51526.

Feng, J., Liu, T., Qin, B., Zhang, Y., and Liu, X.S. (2012). Identifying ChIP-seq enrichment using MACS. Nature protocols *7*, 1728-1740.

Langmead, B., and Salzberg, S.L. (2012). Fast gapped-read alignment with Bowtie 2. Nature methods *9*, 357-359.

Mak, A.B., Ni, Z., Hewel, J.A., Chen, G.I., Zhong, G., Karamboulas, K., Blakely, K., Smiley, S., Marcon, E., Roudeva, D., *et al.* (2010). A lentiviral functional proteomics approach identifies chromatin remodeling complexes important for the induction of pluripotency. Molecular & cellular proteomics : MCP *9*, 811-823.

Rai, K., Jafri, I.F., Chidester, S., James, S.R., Karpf, A.R., Cairns, B.R., and Jones, D.A. (2010). Dnmt3 and G9a cooperate for tissue-specific development in zebrafish. The Journal of biological chemistry *285*, 4110-4121.

Robu, M.E., Larson, J.D., Nasevicius, A., Beiraghi, S., Brenner, C., Farber, S.A., and Ekker, S.C. (2007). p53 activation by knockdown technologies. PLoS genetics *3*, e78.

Walkey, C.D., Olsen, J.B., Guo, H., Emili, A., and Chan, W.C. (2012). Nanoparticle size and surface chemistry determine serum protein adsorption and macrophage uptake. Journal of the American Chemical Society *134*, 2139-2147.

Wong, L., Weadick, C.J., Kuo, C., Chang, B.S., and Tropepe, V. (2010). Duplicate dmbx1 genes regulate progenitor cell cycle and differentiation during zebrafish midbrain and retinal development. BMC developmental biology *10*, 100.
